# A Comparison Between Plant Photosystem I and Photosystem II Architecture and Functioning

**DOI:** 10.2174/1389203715666140327102218

**Published:** 2014-06

**Authors:** Stefano Caffarri, Tania Tibiletti, Robert C. Jennings, Stefano Santabarbara

**Affiliations:** 1 Aix Marseille Université, Laboratoire de Génétique et Biophysique des Plantes, 13009 Marseille, France;; 2 CNRSCentre National de la Recherche Scientifique, UMR 7265 Biologie Végétale et Microbiologie Environnementales, 13009 Marseille;; 3 CEA, Institut de Biologie Environnementale et Biotechnologie, 13009 Marseille, France;; 4 Consiglio Nazionale delle Ricerche, Istituto di Biofisica, 20133 Milan, Italy;; 5 Dipartimento di Biologia, Università degli Studi di Milano, 20133 Milan, Italy

**Keywords:** Plant photosystems, light harvesting complexes, energy transfer, electron transfer, photoprotection.

## Abstract

Oxygenic photosynthesis is indispensable both for the development and maintenance of life on earth by converting light energy into chemical energy and by producing molecular oxygen and consuming carbon dioxide. This latter process has been responsible for reducing the CO2 from its very high levels in the primitive atmosphere to the present low levels and thus reducing global temperatures to levels conducive to the development of life. Photosystem I and photosystem II are the two multi-protein complexes that contain the pigments necessary to harvest photons and use light energy to catalyse the primary photosynthetic endergonic reactions producing high energy compounds. Both photosystems are highly organised membrane supercomplexes composed of a core complex, containing the reaction centre where electron transport is initiated, and of a peripheral antenna system, which is important for light harvesting and photosynthetic activity regulation. If on the one hand both the chemical reactions catalysed by the two photosystems and their detailed structure are different, on the other hand they share many similarities. In this review we discuss and compare various aspects of the organisation, functioning and regulation of plant photosystems by comparing them for similarities and differences as obtained by structural, biochemical and spectroscopic investigations.

## INTRODUCTION

Oxygenic photosynthesis is thought to have begun around 2.4 billion years ago [1] and drastically changed life on earth due to the accumulation of molecular oxygen in the atmosphere and an equivalent reduction in carbon dioxide levels. The first oxygenic organisms, the ancestor of modern cyanobacteria, acquired the ability to oxidise water through the evolution of more ancient not-oxygenic photosystems through a process that is only partially understood [2]. The ability to use light energy to extract electrons from water to generate highly reducing compounds, such as NADPH (E°’~ –0.3), and high energy compounds, such as ATP, which are utilised by the chloroplast metabolism to fix carbon dioxide, and the concomitant possibility to use the newly available molecular O_2_ (E°’~ +0.8) as final acceptor in oxidative reactions made possible the evolution of the most exergonic oxidative metabolism known, that is the aerobic respiration. Almost all forms of life today depend on the ability of photosynthetic oxygenic organisms to convert light energy into chemical energy and to produce molecular oxygen. Photosynthesis also played and plays a major role in the control of atmospheric CO_2_ concentration through carbon fixation,which is also of fundamental importance for life on the planet. Moreover, modern economies are heavily dependent on photosynthetically produced fossil fuels, which contain sunlight energy harvested millions years ago.

Due to the large difference in the redox potential between the electron donor (oxygen in a water molecule) and final electron acceptor during the light phase of photosynthesis (NADP+), the ancestor cyanobacteria had to evolve the capability to use two photosystems working in series in order to be able to accumulate the energy of two photons. These photosystems are called Photosystem II and Photosystem I (PSII and PSI, respectively). They are electronically connected by an intermediate membrane supercomplex called Cytochrome *b_6_f* (Cyt *b_6_f*) [3, 4] and two electron carriers, a liposoluble quinone molecule (plastoquinone) that transports electrons between PSII and Cyt *b_6_f*, and the luminal copper-containing soluble protein plastocyanin, which links Cyt *b_6_f* to PSI.

An ancestral symbiotic event between a cyanobacterium (~1.5 billion years ago) [5] and a eukaryotic cell transformed the first organism into a proto-chloroplast and opened the way to evolution of green eukaryotic photosynthetic organisms (plants and green algae) [5], while other subsequent symbiotic events also allowed the evolution of other types of eukaryotic photosynthetic organisms [6-8]. However, for almost 2 billion years life remained mainly confined in the water and land plants appeared only about 0.5 billion years ago [9]. The first land plants had to challenge new environmental constraints and stresses: an atmosphere rich in oxygen, an environment rapidly fluctuating in terms of light quantity and quality, temperature, nutrients and water.

In green organisms (Viridiplantae), most of the light is absorbed by the photosynthetic pigments chlorophyll *a* and *b*, which have remarkable physicochemical properties allowing efficient light harvesting and ultra fast excitation energy transfer amongst antenna chlorophylls, leading to the quantum and thermodynamic efficiencies which are the highest known. When a photon is absorbed, a chlorophyll is excited to the singlet excited state (^1^Chl*). Apart from energy transfer, one of the principal mechanisms of deactivation of ^1^Chl* is the population of its triplet excited Chl (^3^Chl*) by intersystem crossing (ISC), which involves the inversion of the (excited) electron spin. The excited triplet state of a Chl can interact with molecular oxygen, which is a triplet in its ground state, generating the extremely reactive oxygen singlet species (^1^O_2_*) that can lead to photo-oxidative damaging of proteins, chromophores and membrane lipids. Since ^3^Chl* is populated from ^1^Chl*, the probability of its formation is low under optimal conditions because the singlet excited state population is kept at rather low levels by photochemical conversion, which is very efficient and faster than ^3^Chl* formation. On the other hand, if excitation energy cannot be used for photochemistry, for example when the light intensity exceeds the photosynthetic capacity, ^3^Chl* formation can lead to photo-oxidative stress of the photosynthetic apparatus. Thus, all these factors (a rapidly fluctuating environment and a high reactivity of excited Chls with oxygen) were important for the evolution of the photosynthetic process and its regulations before and during land colonisation.

The two photosystems have a common organisation and are functionally organised in two main moieties: a core complex, containing the reaction centre where the photochemical reactions occur, and a peripheral antenna system that increases the light harvesting capability, but that is also involved in regulation of the photosynthetic process [10, 11]. The core complexes have been well conserved during the evolution, as most of the subunits are similar in prokaryotic and eukaryotic photosystems and only a few are specific to each group [12]. On the contrary, the peripheral antenna system displays great variability, being composed of peripheral associated membrane proteins in cyanobacteria, the phycobilisomes, and integral Lhc membrane proteins in eukaryotic cells. These important topics have been presented in several recent reviews and we suggest reading the following papers for a greater understanding [13-15].

A large body of investigation has been dedicated to the comprehension of photosynthesis in plants over several decades. Although many aspects of the photosynthetic process are nowadays substantially elucidated, several details, specific regulations, and even structural details about photosynthesis in plants are still little known. Hence, in the present review we will focus on the comparison between PSII and PSI of plants. We will discuss about the functioning, organisation, regulation of photosystems under different environmental conditions, by analysing common and specific aspects of each photosystem and by presenting open questions that requires further investigation in order to better understand their functioning.

## 1. PHOTOSYSTEM STRUCTURE

### 1.1. Brief Overview on the Evolution and Structure of the Plant Photosystem Reaction Centers

In the following a brief description of the origin of plant PSI and PSII is presented. Photosystems contain the so called “reaction centres” (RC), the sites where photochemical reactions occur, which are typically divided into families (or groups) depending on the chemical nature of the terminal electron acceptors. Photosystem II has a quinone type reaction centre (also known as Q-Type or type II), while photosystem I has an iron-sulphur (FeS) type reaction centre (or type I). Both families of RC are present in membranes of oxygenic photosynthetic organism. On the other hand, non-oxygenic phototrophic organisms generally harbour either a Type-II reaction centre (i.e., purple Bacteria) or a Type I reaction centre (e.g., green-sulphur or Heliobacteria). From a functional point of view, the cofactors which act as terminal acceptors within Type I reaction centres are more reducing (typical ranges of *E°*= –300/–500 mV) than that of Type II RCs (typical values *E°*= ±30 mV), whereas the standard redox potential of the cofactors which participate in the “donor side” of bacterial RCs are almost the same as that of Type I RCs (in the range of +400/500 mV). Non-oxygenic bacterial photosystems usually operate a cyclic electron transport involving the photosystem and a cytochrome complex, which is analogous to the respiratory complex Cyt *bc*_1_ [3] as well as other diffusible electron carrier proteins, with the most common electron donor to the RCs being small soluble *c-*type cytochromes. This is not the case for oxygenic RCs where the two types of photosystem operate principally, under physiological conditions, in series; the terminal electron donor coordinated by PSII has an *E°*~ +0.9-1 V, which is required to oxidise water (*E°*_H2O/O2_~ +0.8 V), whereas that of PSI is +0.5 V, which is similar to that of bacterial RC.

Photosynthetic reaction centres are most commonly hetero-dimer pigment-protein complexes and this is the case for all known oxygenic photosystems. Structural, biochemical and biophysical analyses reveal a high degree of similarity between the two subunits composing the RC, suggesting that this common organisation originated from an ancient non-oxygenic homodimeric complex [2, 16].

In the past decades crystallographic models of several photosynthetic reaction centres have been presented with sufficient resolution to discern not only the overall structural organisation of the complexes, but also the positions of most of the cofactors. However, whereas crystallographic models have been obtained for both higher plant PSI (3.3 Å) [17] and cyanobacterial PSI (2.5 Å) [18], a high resolution structure is still not available for plant PSII. Nevertheless, due to the great similarity between the plant and cyanobacteria RC proteins, the high resolution structures of the cyanobacterium *Thermosynechococcus elongatus *(2.9 Å) [19] and* Thermosynechococcus vulcanus *(1.9 Å) [20] are usually used as models for the organisation of plant PSII.

Some information concerning plant PSII structural organisation was retrieved from single particle analysis of cryo-electron microscopy at moderate resolution (~17 Å) [21, 22], which allowed some comparison of the overall structural organisation. By comparing the low resolution structure of plant PSII cores [23, 24] to PSII from cyanobacteria, a slightly different organisation of the eukaryotic and prokaryotic reaction centres has however been observed [24, 25]. Moreover, plant PSII core contains specific subunits not present in PSII of cyanobacteria [26].

### 1.2. Structure of the Core Complexes

**Fig. (1):**
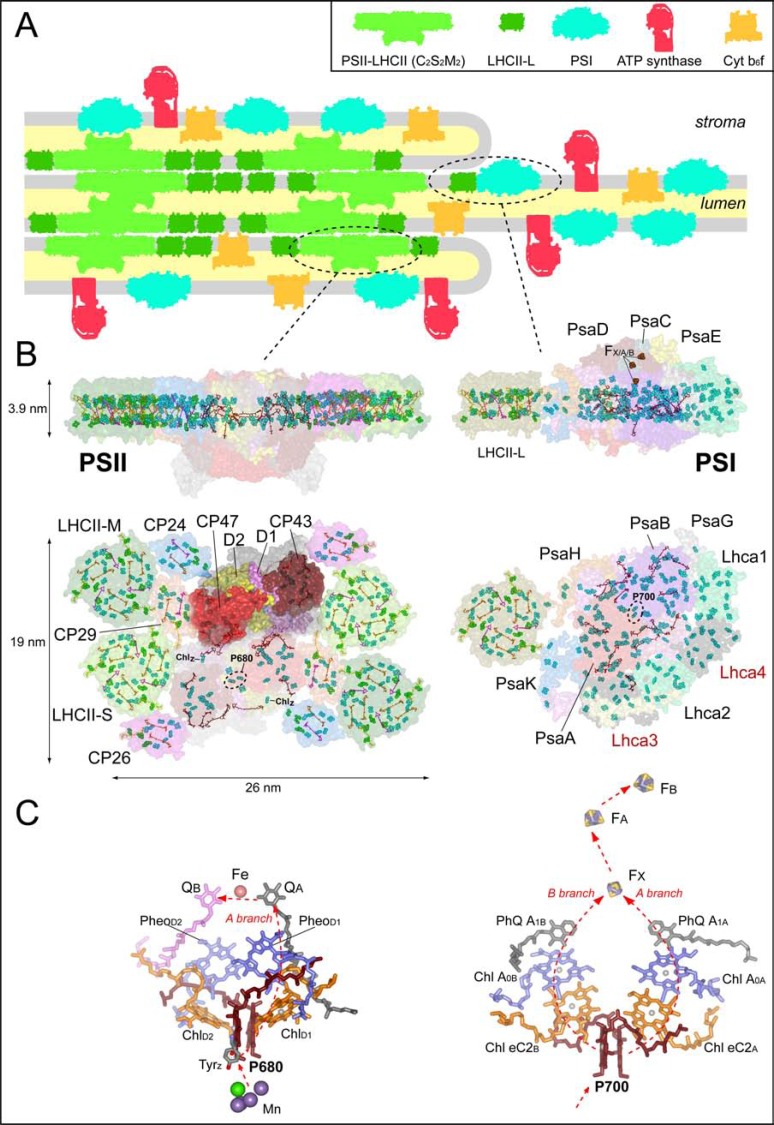
**A**) Simplified model of plant thylakoid membranes. The four photosynthetic complexes involved in the electron transport and ATP synthesis are shown. PSII-LHCII is mainly located in the internal part of the grana membranes. PSI and ATP synthase are located in the stroma-exposed membranes, which are the last layers of the grana and the stroma lamellae. Location of Cytochrome *b6f* is more controversial: this complex is considered evenly distributed in the two kinds of membranes, but highly purified appressed grana membranes (called BBY) do not contain Cyt *b6f* [61, 85, 430]. Thylakoids are dynamic membranes which are subjected to reorganization for the number and size of the stacks, volume of the lumen and location of the complexes. For more information, we suggest referring to the following reviews and references therein [85, 92]. Image modified from [85].** B**) Lateral and lumenal views of the PSII-LHCII supercomplex (C2S2M2) and of the PSI-LHCII supercomplex (as found in State II conditions). PSII-LHCII model has been constructed updating the model in [61], using the crystal structures of the cyanobacterial PSII core [19] (3BZ1 and 3BZ2), the LHCII trimer structure [122] (1RWT) and the recent CP29 structure [124] (3PL9). PSI is based on the structure from pea [17] (2WSC) and the position of mobile LHCII is as in [48]. In the upper of the two monomers composing the dimeric core of PSII, the D1 and D2 reaction center subunits and the internal antenna proteins of the core complex CP43 and CP47 are indicated in color and other core proteins are shown in transparent grey. External Lhc antenna proteins are also indicated: CP24, CP26 and CP29 are the monomeric Lhcb; LHCII-S, -M and -L are the LHCII trimers strongly, moderately and loosely bound to PSII, respectively. Part of the LHCII-L can migrate to PSI under State II conditions [48]. In the PSI model the PsaA and PsaB core subunits are indicated, as well as the Lhca antenna complexes and other subunits visible from the luminal view. Lhca3 and Lhca4 are in red to highlight the fact that these complexes harbor the lowest energy Chls (red forms). The position of Chls and carotenoids is also shown. In the case of the Chls, for simplicity they are represented by only four atoms: the central magnesium and the 4 nitrogen atoms (NA, NB, NC, ND). In PSII and LHCII, Chls *a* are indicated in blue and Chls *b* in green. For PSI, the low resolution of the crystal structure in the Lhca region, where Chls *b* are, does not allow assigning their position and thus all Chls are indicated only in blue. Carotenoids are drawn using the following colors: b-carotene, brown; lutein, orange; violaxanthin, violet; neoxanthin, yellow. Several carotenoids are not resolved in the PSI crystal structure (in particular in the Lhca region and at the interface Lhca-core) and thus they are not shown. The special pairs (P680 in PSII and P700 in PSI) are highlighted with dotted ovals. ChlsZ in PSII RC are also indicated. The FeS center acceptors are only shown for the lateral view of PSI. Note also that the proteins on the PSI side docking LHCII are only partially resolved, thus in this region other peptides (and likely some Chls) do not appear in the crystal structure and in this figure. **C**) Cofactors involved in electron transfer reactions of PSII (left) and PSI (right). Note that a high resolution structure for PSII is not available and thus here it is shown the cyanobacterial RC structure [19] (3BZ1). For PSI, the RC is extracted from the structure of pea [17] (2WSC). P680 is the “special pair” of PSII. Electron extracted from water in the Mn center of the OEC goes through a Tyrosine (Tyrz) to the P680. From excited P680*, electrons follow the “A branch” by reducing in sequence the ChlD1, the PheoD1 (pheophytin), the QA (quinone) and finally the QB (which double reduced and protonated detaches from PSII). An iron atom (Fe) is in between the two quinones. P700 is the “special pair” of PSI. From excited P700*, electrons follow both the “A branch” and “B branch” to reduce an accessory Chl eC2A/eC2B, a second Chl A0A/A0B and then a PhQ A1A/1B (phylloquinone). Electrons from both branches converge on the Fx acceptor and exit from PSI by passing two other 4Fe-4S centers, FA and FB. See text for further discussion on ET mechanism and reaction sequence.

Photosynthetic reaction centres are embedded in the so called “core complexes” (Fig. **1B**). The core of PSII is a multi-subunit complex composed of about 25-30 subunits. The precise number is still unknown, since in recent years advances in purification and mass spectrometry analyses led to the discovery of new subunits, some of which are probably only transiently bound to PSII [26]. Moreover the exact number of subunits appears also to be species-specific. Most of the protein subunits have low (≤ 15 kDa) or very low MW (≤ 5 kDa). Their function is only partially understood and apparently they are mainly involved in PSII assembly, repair and regulation [26, 27]. Yet, most of the chromophores involved in light-harvesting, as well as electron transfer reactions, are bound to four main subunits, known as D1, D2, CP43 and CP47, all of which are membrane proteins containing several trans-membrane helices (TMH). Both D1 and D2, which are homologous, posses 5 TMH each and form a hetero-dimer in which the TMHs are organised in a handshake motif. The D1-D2 complex together with Cyt *b_559_* (composed of the PsbE and PsbF subunits) is often referred to as the PSII RC because it binds most of the cofactors in the photo-catalytic activity of this photosystem (this will be discussed further in the successive paragraph). All together, the D1-D2 complex binds 6 Chls *a*, four of which may be involved in photochemistry, 2 Pheophytins (Chl *a* free base), two β-carotenes, two phylloquinones, iron and the 4Mn-1Ca cluster which is the metallic catalytic core of the Oxygen Evolving Complex (OEC) (Fig. **1B**, **1C**). The Cyt *b*_559_, tightly bound to the D1-D2 heterodimer, is involved in alternative electron transfer processes within the PSII RC [28]. The two large core subunits CP47 (PsbB) and CP43 (PsbC) [29, 30] are bound to the D1-D2-Cyt *b*_559 _complex. Each of these subunits, containing 6 TMH, is associated with one of the RC heterodimer, the binding of CP43 being slightly more labile based on detergent effects [31]. Collectively CP43 and CP47 bind a total of 29 Chls *a* molecules (based on the cyanobacterial structural model) that function as internal antenna and allow excitation energy transfer from the peripheral antenna system to the RC (see below for further discussion on pigment stoichiometry in plant PSII). The Chls *a* composing the internal light harvesting system appear to be organised predominantly in two layers parallel to the membrane plane, and approximately localised in proximity of the luminal and stromal sides of the membrane (Fig. **1B**). On the other hand the cofactors involved in electron transfer (ET) reactions are organised in two parallel chains (or branches) that are perpendicular to the membrane plane (Fig. **1C**). This configuration allows the transfer of electrons across the membrane and the formation of a photochemically generated electrochemical potential. Peripherally associated proteins on the lumenal side are directly involved in water oxidation (PsbO, also called OEE3) or in the optimisation of the process (PsbP and PsbQ, also called OEE23 and OEE17) [32].

The core complex of PSI core is composed by a smaller number of proteins (~15 subunits) than PSII [33]. The large PsaA and PsaB subunits (MW ~80 kDa), which contain 11 TMH each, form a hetero-dimer that binds the vast majority of cofactors for light harvesting (~80 Chls *a *and ~20 β-carotenes) as well as cofactors involved in the electron transfer reactions (6 Chls *a*, 2 phylloquinones and a 4Fe-4S cluster, known as F_X_), with the exception of terminal electron acceptors (Fe-S clusters F_A_ and F_B_), which are bound by the PsaC subunit (Fig. **1B**, **1C**). As in the case of PSII, the Chl *a* molecules involved in proximal light harvesting are organised in two layers parallel to each other and located close to the luminal and stromal sides of the membrane, respectively, whereas the cofactors involved in the ET reactions form two parallel branches, related by pseudo-C2 symmetry, perpendicular to the membrane plane (Fig. **1C**). Excluding PsaA and PsaB, which compose the PSI RC, the other subunits, including PsaC, are of relatively small size ranging from 4 to 18 kDa [18, 33, 34]. These are involved in different processes, such as plastocyanin docking (donor site, PsaF), ferredoxin docking (PsaC, PsaD, PsaE, acceptor side), stabilisation of the LHCI antenna system (PsaK, PsaG) or formation of the docking site for LHCII binding to PSI (PsaH, PsaO) [33]. In cyanobacteria, some of the small subunits are involved in the super-structural organisation of the core complex that will be described in further detail below.

Comparison of the overall arrangement of TMH in the PsaA-PsaB dimer and the D1-D2-CP43-CP47 tetramer reveals substantial similarities in the overall structure of the photosystems, which is also supported by the partial structural homology between the D1 and D2 proteins of PSII (5 TMH each), which are homologous with each other, but which are also related to a domain of the PsaA/PsaB core proteins of PSI (11 TMH each). Similarly, internal antenna CP43 and CP47 (6 TMH) of PSII are similar to each other and are related to a different domain of PsaA and PsaB, indicating an interconnected evolution of PSI and PSII from common ancestral proteins.

The high turnover of D1 subunit [35, 36] might explain the reason why the core pigments of PSII are located on separated proteins (CP43 and CP47) with respect to the reaction center proteins (D1 and D2). D1 is indeed very sensitive to oxidative stresses and plant needs to partially disassemble PSII and substitute D1 at a high rate, while other subunits are recycled [37]. This is simpler and energetically more efficient if the PSII core is composed by modular smaller subunits.

In general no homology exists between small PSI and PSII core subunits, suggesting that these proteins appeared after the evolutionary divergence that generated the ancestors of PSI and PSII reaction centres. It is likely that these subunits originated to optimise the assembly and catalytic activity of PSI and PSII and to adapt to new environmental niches and environmental conditions, such as an atmosphere getting richer in molecular oxygen. Extensive reviews concerning the evolution of photosynthesis have been recently published and we recommend them for more detailed information on these issues [2, 38].

### 1.3. Reaction Centre Structure and Cofactor Arrangement

All the structural models of photosynthetic RCs with sufficient resolution, i.e., that of purple bacteria and cyanobacterial PSII (Type II family), and eukaryotic and prokaryotic PSI (type I family), show a somewhat similar motif in the arrangement of cofactors involved in electron transfer reactions: they are organised in two chains (often also called branches) arranged along a pseudo-C2 symmetry axis and located at the interface of the two main RC subunits. Both of the two cofactor chains is coordinated preferentially by one of the two RC subunits, so that normally the ET chain is referred to accordingly, even though there are also “cross over” in the cofactor co-ordination pattern which is the result of the complex handshake arrangement of the TMH in the RC heterodimer. Thus, limiting the description to oxygenic RCs, each monomer of the reaction centres, PsaA and PsaB in PSI, PsbA (D1) and PsbD (D2) in PSII, contains a similar set of cofactors (Fig. **1C**): a group of at least six chlorines, which are the pigments from which photochemical charge separation and primary electron transfer events are initiated, and two quinone-type molecules, plastoquinones (PQ) in PSII and phylloquinones (PhQ) in PSI, which act as successive electron acceptors. Other cofactors are specific to each ET chain. In the case of PSI, five of the pigments involved in photochemical charge separation in PSI are Chls *a* and the remaining is a Chl *a*’ (the 13’ epimer of Chl *a*), whereas in PSII four are Chls *a* and the remaining are pheophytins. Of the six chlorine pigments, two Chl *a* molecules in PSII and a Chl *a*/Chl *a*’ hetero-dimer in PSI, each co-ordinated by one of the two RC subunits, forms a face-to-face dimer (although the exact geometry is different in the two RCs), whose symmetry axis is almost collinear with the axis of symmetry of the RC. These have been frequently identified with sites of primary photochemical charge separation (primary donor, P), and for this reason are also referred to as “special pairs” and hence assigned to P_680 _in PSII and P_700_ in PSI (the names indicate the maximum absorption in the red region). The exact mechanisms of charge separation both in PSI and PSII are still a matter of debate and will be further discussed below. Of the remaining four chlorines, which are part of the group of pigments active in photochemical charge separation**,** two Chls *a,* each of which is part of one of the two ET chains, are positioned at an angle of 30-45º with respect to the membrane that is perpendicular to the symmetry axis. These Chls are located between the P_680_ dimer and the pheophytins in the case of PSII, and in between the P_700_ dimer and the remaining two Chl *a* molecules, also known as A_0_, in the case of PSI. These Chls *a* are often called “accessory” Chls, as similarly positioned BChls were resolved in the crystallographic model of purple bacteria before solid evidence for their involvement in ET reactions were available from spectroscopic investigations [39]. In recent years, in both oxygenic reaction centres, it has been suggested that the so-called accessory Chls play the role of electron transfer intermediates, possibly being the primary donor [40-44]. On the other hand, there is a general consensus that Pheo in PSII and the A_0 _Chls in PSI act as the primary electron acceptors.

Even though similar “overall” structural arrangements of the cofactors involved in primary charge separation reactions exist in PSI and PSII, it is now solidly established that there are profound differences in the molecular mechanisms of such reactions. In the case of PSII, only one of the two electron transfer branches is used with high efficiency (A branch, Fig. **1C**) and therefore only one of the two Pheo molecules (bound to the D1 subunit) is reduced photochemically. This is known as monodirectional or asymmetric ET. On the other hand, in PSI both ET branches are used with comparable efficiencies so that both the A_0_ coordinated by PsaA (A_0A_) and PsaB (A_0B_) are reduced following charge separation. The latter is known as bidirectional or symmetrical electron transfer [44, 45].

As a result of the monodirectional ET in PSII RCs, mentioned above, only one of the two plastoquinones, Q_A_, which is coordinated to the D2 subunit of PSII, is reduced to the semi-quinone form directly from Pheo_D1. _The remaining quinone, Q_B,_ is reduced to the semi-quinone (Q_B_^–^) by Q_A_^–^ as a result of one charge separation event, and to the fully reduced quinole form Q_B_^2–^ (which is then protonated to become Q_B_H_2_), after a second charge separation event, which is a process known as the two electron gate. Q_B_ represents the terminal electron acceptor of PSII, which in a reduced and protonated form can diffuse out of the RC binding site and acts as a lipophilic electron carrier within the thylakoid membranes. 

On the other hand, in the case of the PSI reaction centre, the PhQ molecules coordinated both by PsaA (A_1A_) and PsaB (A_1B_), are reduced directly from the upstream cofactor in the ET chains, the chlorophylls A_0A/B_^(–)^. Moreover, the PhQs are not the terminal electron acceptors, as their semi-quinone form is oxidised by the iron sulphur cluster F_X_ which is coordinated at the interface of the PsaA and PsaB subunits and is a common cofactor to both ET chains. The electrons are then transferred, sequentially, from F_X_ to the PsaC-bound cluster F_A_ and F_B_, which represent the terminal electron acceptors within the RC. These reduce the soluble electron carrier protein, ferredoxin, and finally NADP^+^ to produce NADPH. Differently from the case of PSII, where two electrons are accumulated by the terminal acceptor Q_B,_ and four oxidising equivalent are accumulated at the donor side at the level of the OEC (where two water molecules are oxidised to form O_2_), so that the system is effectively reset after four charge separation events, in PSI there is no accumulation of reducing/oxidising equivalents within the reaction centre.

**Fig. (2):**
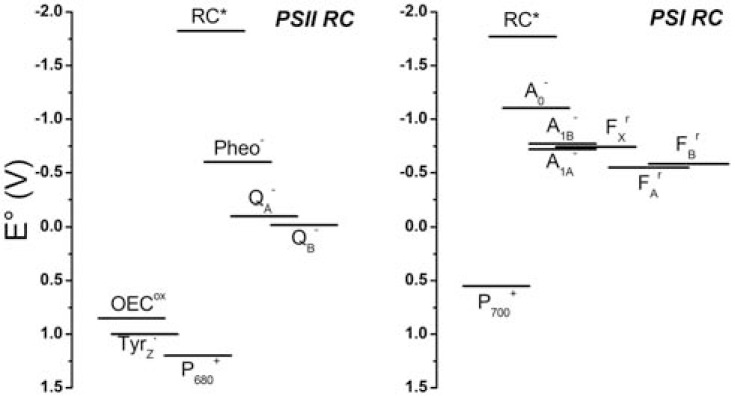
Redox potentials of the cofactors involved in charge separation in PSII and PSI reaction centers.

Another relevant difference, which is linked to the catalytic activity of PSI and PSII, is the redox potential of the chemical species involved in the ET reactions (Fig. **2**). The donor side of PSII is significantly more oxidising (*E°*~ +0.8 V), with the intermediate P_680_^+^ being estimated around +1.2 V (representing one of the most oxidising species in nature), than that of PSI (*E°*~ 0.5 V), i.e., a difference of ~0.3-0.7 V. On the other hand, the acceptor side of PSI is significantly more reducing (*E°*~ –0.5 V) than that of PSII (*E°*~ 0 V), which is about the same difference observed at the donor side. Hence, in both cases an overall potential difference between the donor and acceptor side of ~1 V is established, which is approximately 60% of the energy delivered by a photon corresponding to the lowest excited singlet state transition (~1.7 eV). However, if we consider that the ÄE between the special pair and the first electron acceptors (P_680_/P_680_^+^ and Pheo/Pheo^–^, respectively) is near 1.7 V, the thermodynamic efficiency of the primary photochemistry is ~90%, which is an extraordinary efficiency. Subsequent energy losses are useful to stabilize the primary charge separation and make the reaction directional (low reversibility). A similar situation is found for PSI.

As a result of the difference in operational potential between PSII and PSI, also the donor side of the latter is significantly more reducing than that of PSII; for instance the potential of the primary acceptors, A_0_, is estimated at about –1.2 V, making it one of the most reducing species in nature, whereas the primary acceptors of PSII is estimated at –0.5/–0.6 V, which is about the same as the terminal electron acceptors of PSI. The differences in the operational midpoint potentials are even more obvious when comparing the PhQs (A_1_) in PSI (*E°*~ –0.75/–0.85 V) and the plastoquinone (Q_A_/Q_B_) in PSII (*E°*~ –0.03/0 V). However, whereas in the latter case the difference is also due to the different chemical species, PhQ being more reducing than PQ even in bulk organic solvents, the modulation of the redox properties of Chls *a* induced by the interaction with the protein subunits is rather remarkable and highlights the flexibility of these molecules as redox (as well as light harvesting) cofactors as well as the impressive influence of protein-cofactor interactions in sustaining the catalytic activity of both photosystems.

### 1.4. Oligomeric Conformation

The oligomeric state of PSI has been investigated in various papers in recent years [46-48]. In plants, after purification, other than the most common monomeric form, dimers, trimers and tetramers of PSI have been detected [47]. However, an in depth investigation by single particle analysis of electron microscope images [46, 47] showed that all the oligomers contain PSI units in inverted positions. This configuration is not compatible with the functionality of the photosystem* in vivo*, because electron transfer is vectorial in the membranes, as required to set up an ionic gradient, and indicates that such oligomeric states are very likely artefacts observed *in vitro* after purification. Indeed, it has been demonstrated that oligomeric forms of PSI can be also induced *in vitro* from purified monomeric PSI [48]. Taken together, biochemical and electron microscopy data strongly suggest that plant PSI is a monomeric complex *in vivo*, differently from the case of cyanobacteria, where PSI is found predominantly as a trimer, even though a possible equilibrium between trimers and monomers has been suggested in some cyanobacterial strains [49]. This difference is probably caused by the evolution in plants of the PsaH subunit, which is necessary for LHCII docking, but which impairs formation of trimers in plants. Moreover, it is worth noting that trimerisation of PSI in cyanobacteria is associated which changes in the spectroscopic properties and the appearance of low energy states (red forms) often absorbing/emitting at wavelength longer than 720 nm [50, 51]. In plants, such low energy Chl states are instead associated with the LHCI antenna [52-55], which is not present in cyanobacteria.

**Fig. (3):**
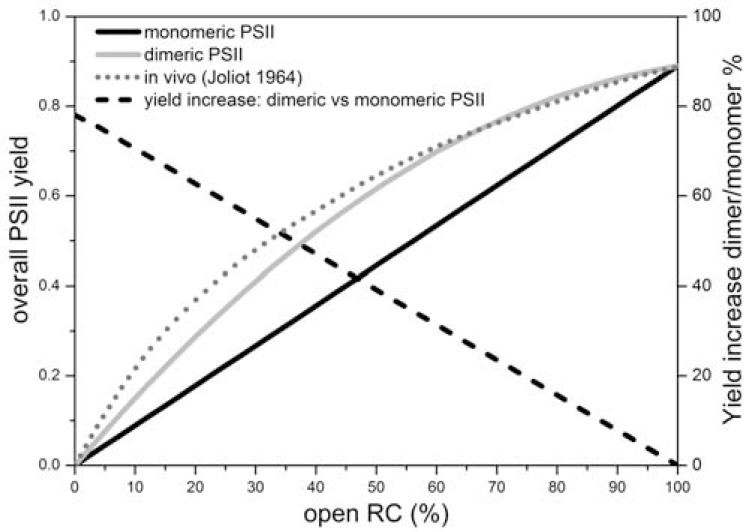
Comparison of the overall PSII photosynthetic yield in dimeric or monomeric conformation in relationship with the fraction of open RC. Photosynthetic yield of open and closed RC is taken accordingly to [62]: 0.89 for open PSII (monomeric or dimeric); 0.78 for dimeric PSII with one open and one closed RC; 0 for closed PSII (monomeric or dimeric). The yield for dimeric PSII is calculated by averaging the yields of all possible PSII dimers in a random population containing a given percentage of open RC. Calculation shows that a dimeric conformation increases overall PSII photosynthetic yield as compared with monomeric PSII. This effect increase when the population of open RC decreases (dotted line). A similar curve has been measured in vivo already long time ago (dotted lined, retraced from [65, 431]) and an even higher convexity of the overall PSII yield was found. This indicates that interconnectivity between PSII dimeric units extends that one between monomers in a single PSII dimer (see text for further discussion).

On the other hand, PSII is generally found as a homodimer (where each monomer is the heterodimeric complex described above associated with Lhc antennas) (Fig. **1A**, **1B**). Even though in a recent report it was suggested that PSII in cyanobacteria is a monomer *in vivo* and dimerisation is induced by delipidation after a detergent treatment [56], most of the findings indicate that both in plants as well as in cyanobacteria, the dimeric conformation is the most common and that monomers should be considered mainly as an intermediate step of PSII assembly and disassembly, which is necessary during its repairing as a result of photoinhibition [35]. This conclusion is supported by the evidence that PSII dimers are observed by electron microscopy analysis in intact thylakoid membranes [57-59], which are purified without the aid of detergents, and that the topography of such dimers matches that of isolated plant PSII supercomplexes [21, 60, 61], which require a detergent treatment of thylakoid membranes for their purification. In both cases, most of the plant PSII complexes are present as dimers. A reason for the dimeric conformation of PSII could be the fact that PSII has a slow turnover (mainly determined by PQ replacement at the Q_B_ site) and in a dimeric conformation there is the possibility of an efficient excitation energy transfer between adjacent RC, thus optimising energy utilisation (Fig. **3**). Modelling of excitation energy transfer in plant PSII using time resolved fluorescence decays kinetics of purified PSII supercomplexes indicates that a dimeric conformation in the presence of one closed and one open RC increases light-harvesting utilization by more than 70% as compared with two separated monomers in the same state (one closed RC/one open RC) [62]. For PSII, which has a low overall turnover of few ms [41], this can significantly decrease the probability to form^ 3^Chl* and harmful singlet oxygen. This effect might be particularly important for PSII because this photosystem is particularly sensitive to oxidative stress (see “photoinhibition” section). As example, it can be calculated that under full sunlight in a temperate environment (i.e, at 2000 µmol photons m^-2^ s^-1^), a monomer is excited about 1 time per ms (in this condition the PSII turnover is much slower than 1 ms due to the accumulation of reduced plastoquinone). Similarly, in the presence of one photo-inactivated D1 protein and before PSII monomerization necessary for repairing, which can be delayed and controlled by core complex proteins phosphorylation [63, 64], the second intact core could use the energy harvested by the Lhc antennas associated to the damaged PSII core.

Moreover, both the analysis of the fluorescence induction [65-75] and singlet-singlet annihilation dependency [76, 77] upon picosecond laser excitation suggest that, *in vivo*, the connectivity is probably extended to more than a single dimer, being likely at least four units (two dimers) capable of sharing the excitation energy. This would not require a structural organisation as tetramer, but simply the possibility of efficient energy transfer between adjacent dimers that is not unlikely considering the tight packaging of PSII supercomplexes (see the next session).

### 1.5. Localization and Organization in the Membranes

Plant thylakoids are organised into two main membrane domains: grana membranes, which are stacked membranes with roughly 26-45 Å between adjacent membranes [59, 78, 79], and stroma lamellae, which are non-appressed membranes exposing the surface to the stroma compartment.

Formation of grana is promoted by different forces [80]. In particular the diffuse electronegative surface membrane charges are masked by cations, to form the so called “electrostatic double layer” [81]. This is the principal reason why cations promote grana stacking, as in the absence of cations the inter-membrane electrostatic repulsion maintains thylakoids separate. Stabilisation of the grana structure, both laterally and vertically with respect to the membrane is achieved mainly by van der Waals forces, which occur principally between the Lhc complexes [82]. The suggestion that entropic forces may be involved [83] does not seem to take into account the fact that photosystem separation, associated with grana formation, leads to a large decrease in the membrane configurational entropy, due to the lateral separation of the two photosystems, which must be considered if this idea is to be considered feasible. As a result of the grana formation, Photosystem I is excluded from the stacked grana regions [84]. It has been suggested that the segregation is due to its bulky stromal side, which does not allow it to enter the stacked regions [85]. The observation that chymotrypsin enters into the intergranal space [86], without grana unstacking, is not in favor of this idea and suggests that other forces might be responsible for PSI exclusion from grana stacks.

The biological function of the lateral separation of the two photosystems in higher plants has long been a subject of debate. It is often thought [87] that the main reason is to minimize energy transfer from PSII to PSI (energy “spillover”), due to the lower energy of some Chls in PSI. Spillover does in fact occur in the absence of cations in plant thylakoids, i.e., in the absence of grana. It should however be underlined that, while spillover is often considered to be an uncontrolled and hence an unfavorable phenomenon, there is evidence that this is not always the case. First of all it is a constant feature of such organisms as red algae and cyanobacteria that perform photosynthesis at efficiencies similar to those of higher plants. Secondly, spillover can be an excellent way of achieving a balanced energy distribution between the two photosystems in a similar way that “state transitions” perform this function in plants (see the section about photosynthesis regulation). This is because the “spillover flux”, observed in isolated plant thylakoids resuspended in the absence of cations, is a function of the oxidation state of the PSII reaction centre. In other words under conditions in which the PSII traps are “open” spillover to PSI is slow, as PSII photochemistry is a kinetically much faster competing phenomenon. Spillover increases as the PSII traps become closed. However, it is probable that photosystem lateral separation, which avoids spillover, has allowed the development of a fine regulation of photosynthesis that would not be possible by simple spillover in condition of PSII closure. Indeed, in plants, the relative energy distribution between the two photosystems is performed by the reversible redistribution of part of the Light harvesting antenna complexes between thylakoid compartments in a phenomenon called “state transitions” [88]. This phenomenon might be particularly important to accurately regulate the activity of the two photosystems under not saturating and fluctuating light conditions in natural environments.

Another important effect associated with grana, which is at the same time fundamental for the formation of thylakoid stacking, is the localization of PSII and its antenna, at elevated density, within the stacked membrane regions. This has been shown to lead to the so-called “PSII connectivity” in which energy transfer occurs between PSII units, a phenomenon which allows energy to be transferred from “closed” PSII units to “open” PSII units [89]. This extended PSII inter-connectivity increases the intra-connectivity between monomers of a PSII dimer discussed previously (Fig. **3**). Indeed experimental observations, in which both oxygen and fluorescence techniques were employed, demonstrated that in the absence of cations (thus of stacked grana) such connectivity is abolished [90, 91]. Since the dimeric nature of the PSII core is unaffected by the presence or absence of cations, this indicates that energy transfer occurs between dimeric PSII units within the appressed grana membranes.

In this context we point out that both “spillover” interruption and energy transfer between PSII dimers, both of which are a consequence of grana formation, have the effect of “favoring” PSII primary photochemical activity. As one of the main challenges facing land plants concerns their survival in the presence of light and oxygen, which, as pointed out above, lead to oxidative damage of the photochemical apparatus (photoinhibition) to which PSII is particularly susceptible, it is not unreasonable to suggest that the granal structure may have evolved as a means of functionally combating the effects of this process.

PSII organization in grana membranes is also important for photosynthetic activity and photoprotection. Recent results suggest an organization of PSII both in random and highly organized array configurations [92]. Reorganization of PSII as well as of Lhc antennas under stress conditions seems important to control PSII activity regulation [93-95], which is necessary for efficient photosynthetic activity or photoprotection (see photoprotection section). Moreover, repairing of damaged PSII requires the migration of damaged cores toward grana margins. Premature degradation of PSII can be controlled by keeping them in the grana region where protease cannot access. In conclusion, due to the highly variable environment to which plants are exposed, the presence of different regions of the thylakoid membranes permitting the spatial segregation of PSI and PSII prevents negative interferences between the two photosystems and allows multiple levels of control of their activities (see also [80] for a review).

## 2. ANTENNA SYSTEM

### 2.1. Overview of the Organization

All photosynthetic organisms, with the exception of Heliobacteria, possess an antenna system that increases optical cross-section of the photosystems. During the evolution of photosynthesis in eukaryotes, the membrane-associated phycobilisomes of the cyanobacteria have been substituted by membrane-integral pigment binding complexes called Lhc (Light harvesting complexes) [13, 14, 96, 97]. All Lhc proteins are encoded by nuclear genes, are homologous to each other and share a similar structural organization. Although all Lhc antennas have three helix spanning regions and coordinate Chl *a*, Chl *b* and carotenoid molecules, each Lhc has a specific pigment content which confers them distinct spectroscopic properties [98, 99].

Accordingly to the nomenclature for core subunits, Lhcb and Lhca are the names used to distinguish PSII and PSI pigment-protein complexes, respectively. In the case of PSII, other names are also commonly used to indicate particular pigment-protein complexes (as CP24, CP26 and CP29 for monomeric Lhcb of PSII; see below) based on the apparent molecular weight on a SDS-PAGE obtained at the time of the first characterizations [100-102]. The Lhc family in plants is normally composed by 6 different Lhcb proteins (Lhcb1-6) and four Lhca proteins (Lhca1-4), each one in a specific position in the photosystem (Fig. **1B**). However, each Lhc complex can have different isoforms. This is particularly evident for the Lhcb1 and Lhcb2 proteins which are encoded by several nuclear genes found in variable but always elevated number in different plant genomes [97, 103, 104]. It is worth mentioning that other genes coding for Lhcb and Lhca proteins, which are still not well characterized from a spectroscopic and functional point of view, are also found in genome analyses. Certain complexes are found in substoichiometric amounts with respect to the core subunits and their function, which has still not been elucidated, might be important under particular environmental conditions, such as under stress (see [105, 106]). This has been proposed for Lhcb4.3, which is part of the PSII antenna (also called Lhcb8 [107]), and for Lhca5, which is part of the PSI antenna [108, 109]. Certain Lhc proteins have been found only in particular plant lineages: for example Lhcb9 in the moss *Physcomitrella patens* [104, 110], suggesting that specific *lhc* genes have evolved recently, probably in order to optimize photosynthetic light harvesting in particular environments. *Physcomitrella patens* is indeed a shade bryophyte (note also that *Physcomitrella* lacks the Lhca4 proteins and there is not a clear distinction between Lhcb1 and Lhcb2 isoforms as in Spermatophytes) [104].

The organization of the Lhc protein around the photosystems is somehow different between PSII and PSI. According to crystallographic model and single particle analysis, in plant PSI, a single layer of Lhca proteins (referred also as LHCI) is bound on one side of the core complex: starting from PsaG (Fig. **1B**), which is considered important (but not indispensable) for the anchoring of the LHCI antenna system [111], the order of binding is: Lhca1, Lhca4, Lhca2, Lhca3. Isolation of single Lhca complexes in intact form has been so far unsuccessful, so that LHCI is typically purified as a “pool” or, at best, in dimeric form. Nonetheless, the properties of the single constituents has been studied in complexes which were reconstituted *in vitro* after heterologous expression in *E. coli* [54, 112-114]. This analysis showed that the properties of the single Lhca are different from each other [53, 54, 114]: in particular Lhca3 and Lhca4 have a far-red shifted absorption/fluorescence spectrum as compared with Lhca1 and Lhca2, due to the presence of a particular environment that gives rise to low energy Chls forms [53], with the most red-shifted form associated with Lhca4. Although initial investigation indicated different properties of the heterodimers composed by Lhca1/4 and Lhca2/3, recent studies in which these complexes have been purified from mutants of Arabidopsis lacking either Lhca1/4 or Lhca2/3 [115] indicate very similar biochemical and spectroscopic properties of the two heterodimers [115], suggesting a functional organization of Lhca in dimers *in vivo* (see also below for further details). It should also be mentioned that in intact PSI, available evidence indicates the presence of even lower energy forms than those present in both isolated LHCI and reconstituted complexes [116].

In the case of PSII, the core is generally found as a dimer surrounded by Lhcb proteins (Fig. **1A**, **1B**). The external antenna system is composed by monomeric pigment-protein complexes that are in direct contact with PSII core and trimeric LHCII, which is in general less strongly associated to it. In particular, Lhcb5 (CP26) is in contact with CP43, Lhcb4 (CP29) is in contact with CP47 and Lhcb6 (CP24) is in contact both with CP29 and a core subunit that is still not clearly identified, but is very likely plant-specific [61, 85]. LHCII, the major antenna complex of PSII, is a heterotrimer composed by Lhcb1, Lhcb2 and Lhcb3 subunits [103, 117-119]. One such trimer is strongly bound to PSII (LHCII-S) and it is in contact with CP43, CP26 and CP29. A second trimer called LHCII-M (moderately bound), which is particularly enriched in the Lhcb1 and the Lhcb3 isoforms [48], is bound more peripherally to PSII and it is in contact with LHCII-S, CP29 and CP24. Biochemical studies indicate that up to four trimers per PSII can be found *in vivo* [117, 120]. However, the position of these two additional LHCII trimers, which are loosely bound (LHCII-L), is still not clearly defined. The weaker binding of LHCII-L trimers to PSII seems important for the reversible transfer of these complexes from PSII to PSI allowing them to function as PSI antenna, thus adjusting the optical cross absorption section of the two photosystems [48].

The dimeric conformation of PSII was generally considered necessary for Lhc antenna binding [85], but investigation on purified PSII particles with different antenna sizes showed that monomeric PSII can still bind stably both CP26 and LHCII-S [61]. On the other hand, results in [61, 121] show a low amount of particles binding of CP29, CP24 and LHCII-M to one monomer of a dimeric PSII, suggesting that the same antenna complexes can bind to a single monomeric PSII. However it is very likely that such a particle (monomeric PSII-CP24-CP29-LHCII-M), is not very stable and indeed it has not been visualized yet.

### 2.2. Properties of the Individual Lhc Complexes

High resolution structural models of antenna complexes have been obtained only for LHCII (~2.6 Å) [122, 123] and more recently for CP29 (2.8 Å) [124]. LHCI proteins have not been crystallized in isolated form, but some structural information can be retrieved from the structural model of the PSI complex [17]. However, in the Lhca region of the supercomplex the resolution is lower than the overall 3.3 Å resolution of the entire complex, thus preventing a detailed description of the organization of these antenna complexes. Nevertheless thanks to close examinations of the data from the crystal structures [17, 122-124], from biochemical analyses [52, 103, 115, 125-128] and from analysis of gene sequences, a quite well defined description of the pigment content and the putative position of the chromophores in each antenna complex can be obtained (Table **1**). LHCII binds 14 Chls and 4 carotenoids: the structure from pea [122] and spinach [123] indicates 8 Chls *a* and 6 Chls *b* (Chl *a/b* ratio of 1.33), two luteins in two internal binding sites (L1 and L2; in L2 substoichiometric amounts of violaxanthin are also found according to biochemical data [129]), one neoxanthin in a specific N1 site [130, 131] and mainly violaxanthin in the peripheral V1 site [126, 129]. However, it is worth underling two points that have received little consideration: i) the Chl *a/b* ratio of LHCII from different species can be somehow different; for instance, Chl *a/b* ratio of maize LHCII is ~1.5 [129] or even higher (1.7-1.8) under particular growth conditions [132], suggesting that different Lhcb1-3 isoforms have at least one Chl binding site with different affinity for Chl *a* and *b* or have sites with mixed occupancy; it is also possible that the pigment stoichiometry can be controlled during the assembly* in vivo* in response to environmental pressures; ii) the V1 site, which is considered to bind mainly

violaxanthin under low-moderate light, as found in spinach [126] and maize [129], in certain species such as Arabidopsis it is occupied preferentially by lutein rather than violaxanthin [133]. In LHCII, this is the only site capable of exchanging violaxanthin with zeaxanthin synthesized by de-epoxidation of violaxanthin during the operations of the xanthophyll cycle under high light [129]. It is not clear if this site is directly involved in thermal dissipation of excess energy (Non Photochemical Quenching, NPQ; see section photoprotection) or if it is just a reservoir of violaxanthin promptly made available for de-epoxidation. At the same time, it is worth noting that both zeaxanthin and lutein are important for the establishment of Non Photochemical Quenching in plants, as reviewed in [134].

The recent crystal structure of CP29 at 2.8 Å resolution [124] indicates that this complex binds 3 carotenoids and 13 Chls (8.5 Chls *a* and 4.5 Chls *b*, the non-integer number may indicate the presence of a site with mixed occupancy), a number higher than the typical value of 8 previously proposed both for the purified and the *in vitro* reconstituted complex [98, 135, 136]. CP29 differs from LHCII in that CP29 lacks a peripheral V1 site and also differs in the position of some Chls binding sites: pigments are not found at positions equivalent to Chl b601 and Chl b605 in LHCII, whereas a specific site, called Chl a615, is present in CP29. The structure of CP26 is not available, but from biochemical studies and sequence homology, it is likely that this complex has a Chls *a/b* ratio of about 2 [98, 131, 137] and a similar amount of Chls (13-14 Chls), which is similar to that observed in the CP29 structural model but higher than that previously proposed [136]. It has also been proposed that CP26 has a V1 site as LHCII [138], thus binding four carotenoids per polypeptide. However, highly purified CP26 by isoelectrofocusing has at most 3 carotenoids in site L1, L2 and N1 as in the case of CP29 [137]. Since pigments can be lost during purification, especially if weakly bound to the complexes, such discrepancies deserve further investigation, particularly since xanthophylls play an important role in the regulation of NPQ. CP24 is a complex somewhat different from all the other PSII monomeric antennas. It is found only in the land plant lineage (CP26, CP29, LHCII are present in green algae), suggesting a more recent evolution [13, 104]. Moreover, it has unusual pigment binding properties: CP24 is the antenna with the lowest Chl *a/b* ratio (~1), binds 10-11 Chls and only 2 carotenoids in site L1 and L2 according to biochemical studies [128].

The characteristics of the single Lhca complexes are less elucidated than for the Lhcb complexes, mainly due the difficulties of purifying intact monomeric native complexes. Such difficulties are due to the very similar biochemical properties of the individual Lhca, which hamper the purification of the single complexes, and to the strong interactions both between Lhca complexes and between Lhca and the PSI core. This obliges to use relative harsh detergent treatments for their purification [139] and can cause pigment loss. However, thanks to the use of *in vitro* refolded complexes from recombinant apoproteins and purified pigments [53, 54, 114], and thank also to the purification of the Lhca1-4 and Lhca2-3 dimers from mutant plants of Arabidopsis [115], a good description of the Lhca complexes has been obtained. Despite differences in the monomeric Lhca [54, 114], each dimer has remarkably similar properties, as both dimers have a Chl *a/b* ratio of ~3.7 and a Chls/Cars ratio of ~4.6-4.8 [115]. By assuming a Chl content of 28 molecules per dimer [111], this means that 22 Chls *a*, 6 Chls *b* and 6 Cars are present in each dimer. According to data on recombinant Lhca proteins (which probably contain less pigments than native complexes), it is likely that 4 Chls *b* molecules are located in Lhca2 and in Lhca4, and only 2 in Lhca1 and Lhca3. It is however clear, also from the analysis of the whole Lhca pool [114], that the Chl *b* content is lower in Lhca complexes as compared with Lhcb complexes.

Lhca proteins bind lutein (~3 molecules per dimer), violaxanthin (1-1.5 molecules per dimer) and β-carotene (~1 molecule in the Lhca1-4 dimer and 2 molecules in the Lhca2-3 dimer). In particular, since in recombinant complexes β-Car is present only in Lhca3 [54], this suggests that the binding site of this carotenoid is stabilized only after dimerization of the Lhca complexes.

An important difference between Lhcb (excluding CP24) and Lhca proteins is the absence of neoxanthin in the latters. Neoxanthin in Lhcb has been suggested to play, directly or indirectly, an important role in regulation of light harvesting and photoprotection. In particular, it has been suggested to act as a superoxide anion scavenger [140], to be involved in the control of LHCII trimer-trimer interactions [141], as well as to undergo molecular rearrangements which can be used as spectroscopic markers accompanying the onset of NPQ [142-144]. The fact that PSI does not contain neoxanthin could be either due to the fact that ROS scavenging around PSI can be efficiently performed by the stroma soluble enzyme superoxide dismutase, which, on the contrary, cannot enter in the highly appressed grana where PSII is localized (Fig. **1A**).

Another difference between Lhca and Lhcb proteins is the strength of their association to the respective core complexes: while Lhcb proteins can be relatively easily separated from PSII by soft detergent treatments with negligible pigment loss [129], Lhca are more tightly bound to PSI and their separation from PSI requires harsher biochemical protocols that cause some pigment loss and alterations of the spectroscopic properties. The higher strength of the binding between the Lhca complexes and the core of PSI could be due to the presence of “gap” and “linker” Chls located at the interface of these moieties and, probably, also due to the presence of linker carotenoids, as proposed in [111, 145].

The different binding strength of the Lhcb and Lhca complexes to the respective photosystem cores is also functional to the different flexibility of these antenna systems. PSII antenna system is largely reorganized as a response to long- and short-term changes in light intensity: under prolonged high light, the quantity of the LHCII trimers is reduced (in particular of trimers L and likely M), as well as the quantity of CP24 [106]; on the contrary, the quantity of Lhca complexes is relatively constant irrespectively of growth conditions [106]. Moreover, it has been also proposed that the Lhcb antenna system is partially reorganized in order to activate the thermal dissipation of the excess absorbed energy [93, 94] in response to short-term (high) light stress. The modular and flexible arrangement of PSII subunits, including the antenna complexes, can also be functional to the relatively rapid protein turnover of some of the RC subunits of this photosystem compared to PSI. Whereas LHCII turnover is not as rapid, this will represent a simple strategy favouring the disassembly of the supercomplex, especially when damaged, which can then be reassemble minimizing *de novo* protein synthesis, which is a costly process from the metabolic point of view.

In the case of PSI, the major mechanism to adjust the antenna size in response to environmental stimuli seems to be the reversible binding of LHCII trimers under light exciting preferentially PSII in a phenomenon called “state transition” (see section “Regulation of light harvesting capacity”). However, there is some evidence that LHCII binding to PSI is not restricted to moderate light conditions when state transitions are activated [146-148], but also to higher light [106, 149, 150], suggesting that LHCII can be considered an intimate PSI antenna. This is also supported by a recent analysis on the purified PSI-LHCII complex from which it was revealed that the binding of mobile LHCII to PSI is stronger than its binding to PSII, and that excitation energy transfer to PSI appears to be faster than to PSII when the same LHCII is bound to it [48].

### 2.3. Pigment Content in PSI and PSII

Photosynthetic pigments, chlorophylls and carotenoids, are indispensable for light harvesting, excitation energy transfer between photosynthetic subunits, charge separation and photoprotection. In this section we describe the pigment content in PSI and PSII (Table **1**).

First of all, a clear difference exists between the pigment composition of the core complexes and the external Lhc antennas: the core complexes of both photosystems bind essentially only Chl *a* and β-carotene molecules, while the external Lhc antenna complexes also bind Chl *b* and xanthophyll (oxygenated carotenoids).

The high resolution structure of PSI from pea shows that plant PSI binds at least 173 Chl *a* and *b *molecules [17]. In the structure it is not possible to identify the Chl species (*a* or *b*), but biochemical analysis on purified PSI indicate that PSI has a Chl *a/b* ratio in a range of 8.2/9.7 and thus binds approximately 154/157 Chls *a* and 19/16 Chls *b* [48, 109]. These Chls are bound to the Lhca proteins (both Chls *a* and *b*), to the core complex (only Chls *a* for a total of ~100 chls), and between these moieties (both Chls *a* and *b*). The latter represent the so called “linker” Chls (between Lhca monomers) and “gap” Chls (between Lhca and PSI core) and probably play an important role in excitation energy transfer between Lhca antennas and from Lhca toward the PSI core [17, 111, 145]. Biochemical analysis indicated that PSI binds approximately 33/34 carotenoids [48]: ~12 Cars are bound to Lhca proteins and also probably at the interface between the Lhca and the core complex [17, 145], while the others are bound to the core (22 β-car). During state transitions, a subset of LHCII, the major antenna complex of PSII, moves and binds to PSI in order to increase its antenna size (as shown in Fig. **1**). It can then be estimated that the PSI-LHCII supercomplex contains ~215 Chls and 45/46 Cars. From these values, it can be estimated that the Lhc antenna system contributes ~32-38% to the total PSI absorption in the visible region (considering the gap and linker Chls either as part of the core complex or of the antenna system, respectively). For the PSI-LHCII supercomplex, the value for absorption by the Lhc antenna system increases to ~46-50%.

**Fig. (4):**
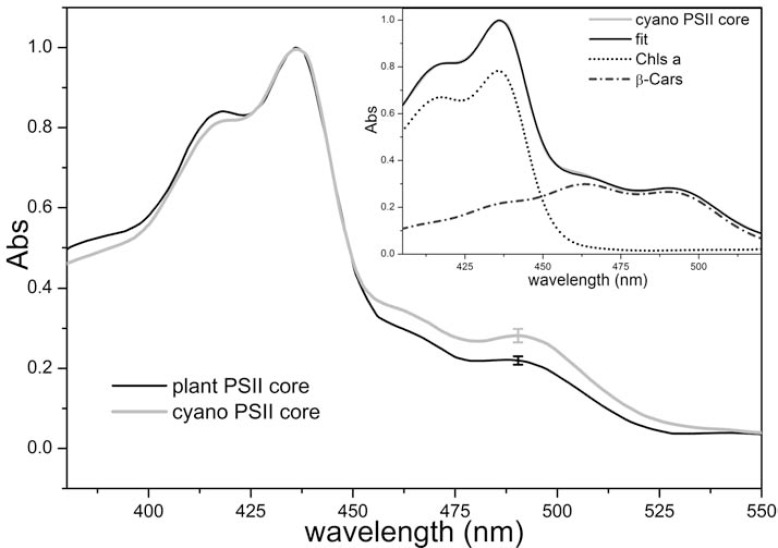
Comparison of spectra of PSII cores from plants and cyanobacteria. The presented spectra are the average of various preparations retrieved from the literature. In the case of plant PSII, 3 spectra have been used: an Arabidopsis PSII core (Caffarri, unpublished), a tobacco PSII core [432] and a spinach PSII core [433]. In the case of cyanobacterial PSII, 5 spectra have been used: three PSII core preparations from *Synechocystis sp.* PCC 6803 (Tibiletti, unpublished; [434, 435]); two PSII core preparations from *Synechococcus elongatus* [152, 436]. Spectra have been normalized at the maximum absorption in the Soret region and then averaged. Standard deviations are indicated in the b-carotene absorption region. In the inset, the deconvolution of the absorption spectrum of the cyanobacterial PSII core, using the individual spectra of Chl *a* and b-Car in acetone 80%, is shown as example. The Chl *a* and b-Cars spectra here shown are the sums respectively of four Chl *a* and two b-Cars spectral forms, each one shifted to a different extent in order to optimize the fit.

For plant PSII, the absence of a high-resolution structural model of either the core or the PSII-LHCII supercomplex does not allow a definitive conclusion about Chl and carotenoid content. At the same time, it is generally accepted that, due to the high conservation of core complex subunits between cyanobacteria and plants, the PSII core of land plants bind the same amount of pigments, i.e., 35 Chls *a* (RC: 6; CP47: 16; CP43; 13) and 11-12 β-carotenes as found for the cyanobacterial core [19, 20]. However, it should be noted that a higher amount of Chls has been suggested to be bound to eukaryotic PSII cores as compared with cyanobacterial cores [151-153]. To further investigate this point, we analyzed various published absorption spectra: the average spectra for plant and cyanobacteria PSII cores show that a difference might exist (Fig. **4**). Moreover, the comparative analysis of PSII particles having different antenna sizes [61, 62] suggested that one b-carotene molecule could be lost during purification of the plant PSII core as compared to the core inserted in PSII-LHCII supercomplexes. Considering that it is unlikely that an intact plant PSII core binds less pigments (both β-carotenes and Chls *a*) than a cyanobacterial one, since they are bound to highly conserved proteins, this suggests that plant PSII core would bind 11-12 β-carotenes as well. By calculating the pigment content using either the absorption spectra in (Fig. **4**) or the HPLC data on plant PSII particles (Caffarri, unpublished), we find that the plant PSII core would bind either 42.5±0.5 or 46.5±0.5 Chls by normalizing to 11 or 12 β-carotenes respectively, which is 7 to 12 more Chls *a* with respect to those present in the same complex of cyanobacteria. This would imply that the specific plant PSII core subunits (such as PsbW and PsbR) might be involved in the binding of some pigments or that plant CP43 and CP47 are able to bind a higher number of Chls than the cyanobacterial homologues. Considering that the PSII antenna system in plants is located in the membrane and that energy transfer from Lhc to the core must follow different pathway as compared with phycobilisomes in cyanobacteria, it is reasonable to suggest that specific Chl binding sites/proteins have evolved for this reason (for instance, Chls located in the core subunits next to CP24 might allow direct energy transfer from this monomeric antenna). A high resolution structure of plant PSII would help shed light on this issue and favour more in depth investigation concerning the functionality of plant PSII.

Moreover, since PSII has a peripheral antenna system that is much more flexible and dynamic than that of PSI [106, 145, 154], which means that the peripheral Lhc antenna system can be increased or decreased accordingly to the environmental conditions, a precise stoichiometry of the pigments bound to PSII cannot be given. However, different PSII supercomplexes can be purified and biochemically characterized [61]. The largest purifiable PSII particle is a dimeric supercomplex containing two copies of each monomeric antenna (CP24, CP26 and CP29) and four LHCII trimers, and is called C_2_S_2_M_2_ (two Cores, two trimers S, two trimers M) (Fig. **1B**) [60]. Accordingly to the Chl and Car content in the core and in each Lhcb complex previously described and on the basis of HPLC pigment analysis (Caffarri, unpublished), the pigment content of the Arabidopsis C_2_S_2_M_2_ dimeric complex is of ~320 total Chls with a Chl *a/b* ratio of ~2.2 and ~86 Cars. Considering that up to 4 trimers (S, M and two loosely bound L trimers) per monomeric PSII can be found *in vivo* under non saturating light [117], this means that up to ~490 Chls (~320 Chls *a* and ~170 Chls *b*) and ~134 Cars constitute the pigment system of a dimeric PSII. It can be further estimated that the absorption by the Lhc antenna system contributes between 73% and 82% of the total PSII absorption in the visible region (calculating for a C_2_S_2_M_2_ complex and for a C_2_S_2_M_2_L_4_ complex, respectively), a much higher value than that retrieved for the Lhca antenna of PSI.

As can be seen in (Fig. **1B**), most of the chlorophylls and carotenoids in PSII are indeed located in the antenna system. By calculating a PSII volume of about ~1900 nm^3^ (26x19x3.9 nm, Fig. **1B**), it is possible to find that the average Chls concentration in PSII is ~0.28 M (in the Lhc antenna system and in the core, respectively ~0.33 M and ~0.17 M). In PSI, which has an oval shape of ~15x20 nm, the Chl concentration is ~0.32 M. Free chlorophylls in solution exhibit a “concentration quenching” even at much lower concentrations than these, as discussed in [155]. This shows how the protein environment is indispensable to organize spatial distribution of Chls and optimize excitation energy transfer with minimal loss of energy. However, it is possible that small conformational changes of photosynthetic proteins can cause the formation of quenching centers, as proposed in different hypotheses concerning the induction of NPQ (see photoprotection section).

**Fig. (5):**
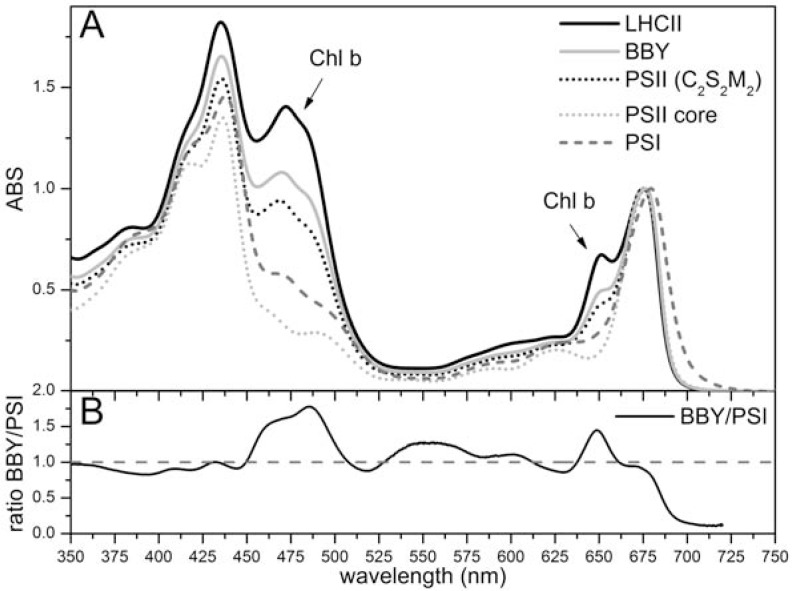
**A**) Absorption spectra of different photosynthetic complexes: “LHCII” is a purified trimeric LHCII as in [129]; “BBY” are purified grana stacks [61] (this spectrum is measured after mild solubilisation of the membranes to avoid light scattering); “C2S2M2”is the largest PSII-LHCII supercomplex purifiable from Arabidopsis [61]; “PSII core” is a purified Arabidopsis PSII core complex (Caffarri, unpublished); “PSI” is a purified PSI-LHCI supercomplex [48]. The presence of increasing amounts of LHCII trimers (as LHCII-L in BBY membranes) results in a clear increase of absorption in the regions where Chls *b* contribution is dominant (475 and 650 nm), because of its larger stoichiometric abundance in LHCII. **B**) Ratio between the spectra of BBY (PSII membranes) and PSI after normalization at the same total absorption in the visible region. The ratio provides a good indication of the wavelengths exciting preferentially either PSII or PSI.

It is also interesting to note that the large amount of Chls *b* in PSII as compared with PSI, as well as the particularly low energy level of certain Chls *a* in PSI, are at the origin of a different absorption spectrum between the two photosystems (Fig. **5**). This can cause an unbalanced absorption by the two photosystems under lights enriched in particular wavelengths. The “state transitions” phenomenon [156-159], discussed later, is an important mechanisms allowing balancing the absorption between the two photosystems with a regulation acting in few minutes.

### 2.4. Absorption Properties of Photosystems and Red Forms

As discussed above and shown in (Fig. **5**), it is well known that the absorption spectra of PSI and PSII display substantial differences, particularly in the Qy absorption region. While the bulk antenna of PSI has maximum absorption close to 682 nm, the corresponding maximum of PSII is somewhat shorter (677 nm). PSII has a prominent Chl *b* peak near 650 nm, which is much smaller in PSI, due to the higher Chl *a*/*b* ratio of PSI with respect to PSII. However the principal, and most interesting difference, is associated with a small number, approximately 8-10 [160] of low energy chlorophylls in PSI (red forms), which absorb at wavelengths above those of P_700_ and which are normally absent in PSII. These are mostly associated with the external antenna complexes Lhca [53]. Over 80–90% of excited states are associated with the red forms at thermodynamic equilibrium [160, 161]. This means that energy must be transferred "uphill" from the red forms in order for photochemical trapping to occur and this is achieved by thermal activation [116]. It is therefore surprising, at first view, that PSI performs primary photochemistry much faster than PSII, in which uphill energy transfer is almost absent (see energy transfer section). Moreover, recent data show that the trapping rate in PSI increases from about 18 ps at emission wavelengths characteristic of the core antenna, to about 80 ps across the low energy emission band [116].

Thus energy transfer in PSI is slowed down by the red forms, which excludes any possibility that their presence in PSI is for energy transfer and photochemical trapping reasons. The problem thus arises as to the function of these low energy chlorophylls. Basically two suggestions have been made concerning this aspect: i) they may have a function in photo-protection in PSI. This idea seems to be derived in relatively recent times from the study by [162] who considered that the red forms have a low fluorescence yield and could therefore be associated with a quenching state, possibly involved in photoprotection; subsequently this claim was disputed by [116, 163] and it would therefore seem that this idea can be rejected; ii) they may have a light harvesting role under conditions of "shade light" (leaves which are within or under a vegetation canopy where the light environment is enriched in wavelengths above 700 nm). This suggestion, initially made in general terms by [164] and [165] was examined in detail by [166] who calculated that under "shade light" the low energy forms of PSI may account for up to 40% of total chloroplast absorption. This observation led to a ready explanation of the high levels of photosystem II antenna synthesised by plants exposed to shade light [167]. Thus the PSI red forms lead to a greatly increased optical cross section of PSI under canopy shade and in order to attain a balanced energy distribution between the two photosystems a large amount of PSII antennas is synthesised [166].

As discussed, the low energy chlorophylls of PSI are an interesting characteristic of this photosystem and would seem to have evolved in order to permit absorption just outside the main absorption band in order to harvest light in the 690–750 nm region under "shadelight" conditions. We will discuss shortly the photophysical properties of these unusual chlorophylls, as they are known at the present.

Over the years it has often been suggested that the large bathochromic shifts could be explained as the low energy transition of an excitonic dimer [168, 169]. This suggestion was subsequently demonstrated to be correct [170-172] with the reported Coulombic (matrix) interaction energy being high (280–335 cm^-1^), indicating strong chlorophyll-chlorophyll interactions. These values, for bulk antenna chlorophylls, are typically around 30 cm^-1^ [173]. As a result of this strong coupling within the dimers the band shapes are extremely broad at room temperature, with reported values for the FWHM in the range of 30–55 nm [174, 175] whereas for bulk antenna chlorophylls the FWHM is usually around 10–12 nm. This extraordinary band broadening is thought to be due the excitonic dimer forming a charge transfer complex, with a high intrinsic dipole moment, which leads to strong electron phonon coupling [175]. One should note that it is the so-called optical reorganisation energy (Sν_m_), where S is the electron-phonon coupling strength to phonons of mean frequency ν_m, _which leads to the thermal band broadening (homogeneous broadening). In the case of the very broad fluorescence emission band at 735 nm of plant PSI, the value of Sν_m_ is expected to be ≈ 250 cm^-1^ and the Stokes shift has the extraordinary value of 25 nm [176]. The absorption origin bands of these low energy chlorophylls can be considered to be in the approximate range of 701–710 nm [175, 176].

## 3. ENERGY TRANSFER AND PHOTOCHEMISTRY

### 3.1. Excitation Energy Transfer

As discussed above, the main function of photosynthetic antennas is that of increasing the probability of absorption of incident photons and ensuring that the excitation energy is efficiently transferred to the photochemical reaction centres, which typically represents only a small fraction of the total chromophores bound to the photosystems. As presented before, the size of the overall antenna of plant PSI-LHCI and PSII-LHCII supercomplexes, considering both the proximal and distal chromophore, is in the order of 170-240 Chls (*a*+*b*) molecules, whereas the cluster of pigments composing the photochemical reaction centre comprises only a small number of molecules. Another strategy developed by photosynthetic organisms to increase the optical cross-section of the antenna, is the modulation of the Chl absorption as a result of the interaction with the protein environment (e.g., [99, 177-179]). Since chromophores are bound at specific sites in each Chl-binding complex, either of the proximal or the external antenna, the spectroscopic characteristics of each bound chromophore are tuned by these specific interactions. Probably the most relevant factors are the axial coordination of the central magnesium atom in Chls, as well as axial coordination to keto-carbonyl groups of the peripheral porphyrin ring and the hydrophobicity of the binding niche. These interaction leads to subtle distortion of the chlorophyll planes, which modify the spectroscopic properties of the bound chromophores [173, 180]. Furthermore, interactions between different chromophores, or cluster of chromophores, also lead to changes in the transition energy as well as the intensity of the transition due to exciton splitting, which also contribute to increasing the energy spread of the antenna pigments [181, 182]. Generally, especially for the case of a whole supercomplex, it is not possible to distinguish the properties of each individual Chl, yet they can be grouped into the so-called “spectral forms”. The most pronounced effect of the protein-chromophore interaction is observed at the level of the lowest energy transitions (*Q*_y_) of Chl *a* that, when bound to photosynthetic complexes, spans a rather large range, with spectral forms observed in the 660 and ~710 nm interval [99, 139, 177-179]. The most red-shifted forms (absorption maxima above 700 nm) are localised in PSI, predominately in the Lhca antenna complement in the case of land plants [54, 115, 116, 139, 171, 172, 176, 183].

For efficient excitation energy transfer to the reaction centres, the transfer of the excited state either in between pairs of monomeric or in between clusters of chromophores needs to be significantly faster than the singlet excited state de-excitation process. It has been generally considered that the dominant mechanism involved in the singlet energy transfer in the antenna, at least amongst Chls, is incoherent inductive resonance process, also known as the Förster transfer mechanism [181, 184-188]. The principal conditions for a fast transfer rate, according to this mechanism, are a good overlap between the emission spectrum of the donor and the absorption spectrum of the acceptors, the geometrical orientation of the transition dipole moments and, crucially, the distance between the donor and the acceptor molecules, since the excited state transfer rate has an inverse sixth power dependence for this parameter. It has been shown that Förster transfer can be efficient even over relatively large distances, exceeding 20-40 Å. Such a range of effective distances exceeds the average distance amongst nearest neighbour chlorophylls in photosynthetic antenna: from structural information, both for the core as well as for internal antenna pigments, this average inter-pigment distance is in the order of ~6-8 Å [17, 18, 20, 122]. Moreover, there is in general a good overlap between the absorption and emission of Chl spectral forms, taking into consideration that they can be considered, to a first approximation, randomly distributed in the antenna array. In the simplest picture, it is possible to represent the energy transfer process in the antenna as a random walk through the different pigments sites that, being positioned at precise locations within the antenna complexes, compose a sort of quasi-regular lattice [75, 189-192]. Thus, starting from the excitation energy being located in any given antenna site, by an essentially random hopping process within the lattice, it will eventually reach the photochemical reaction centre, where charge separation occurs with a certain efficiency. It is possible to envisage two limiting cases describing energy migration and excited state photochemical conversion in a photosystem: the first is that photochemistry is much faster than the energy migration to the photochemically active pigments (purely diffusion-limited case); the second is that energy migration is much faster than photochemical trapping (purely trap-limited case). In real systems neither of these two extreme cases occurs so that both processes, excited state diffusion and the photochemistry, contribute in determining the effective kinetics of energy conversion. Still, one of these two processes might have a “dominant” effect, so that a photosystem could be described substantially as either trap or diffusion limited and this has been the source of much debate in the literature [42, 43, 62, 116, 162, 193-222]. In general, photochemical “trapping” from the reaction centre excited state is in (kinetic) competition with the transfer of excitation energy back to the antenna bed. Thus the excited state can, in principle, be transferred both into and out of the reaction centre several times before primary charge separation occurs, unless the rate of photochemical trapping is much faster (several order of magnitudes) than the excited state “hopping” time. An important consequence is that photochemical reactions take place from the equilibrated excited state of the photosystem, and hence of the reaction centre, although small deviations from the pure equilibration population are possible for the reaction centre pigments due to the proximity of these pigments to the photochemical excited state quencher.

In these terms, the time by which the excited state reaches the reaction centre was demonstrated to scale with the number of sites in the antenna matrix, for the simple case of an isoenergetic system. The increase of the overall excited state migration time, which is proportional to the antenna size, will affect the overall kinetics of photochemical conversion. Although elegant mathematic description of an isoenergetic antenna have been derived under certain assumptions [75, 189-192, 223], this linear dependency of the (overall or mean) excited state transfer time, as well as the yield of the process can also be rationalised on intuitive ground. Since each step between nearest neighbours will proceed at a certain rate, in competition with radiative (fluorescence) and non-radiative (^3^Chl* formation and heat dissipation) decay processes and since a large antenna size will be associated with an increasing number of steps in the random walk occurring before the excited state is finally trapped photochemically, the larger the number of steps, the higher the probably that the excited state will be dissipated non photochemically. At the same time, the more extended the random walk network, the longer the time the excited state resides in the antenna bed rather than in the reaction centre pigments. For a real photosystem, in which the pigment sites are not isoenergetic, this situation is more complex. However in the case of PSII, where the energy difference between the core complexes and the whole of the Lhc antenna complement has been reported to be only a fraction of the thermal energy at physiological temperatures [99] due to presence of Chl *b* in the external antenna, the isoenergetic approximation may be of use. In the case of PSI, due to the presence of low-energy spectral forms [139], the energy spread between the core complex and the external antenna is larger and the isoenergetic approximation is not valid. Moreover, another deviation from the ideal lattice scenario encountered in real photosystem is that the pigments are not organised in a regular and periodic configuration, so that kinetic bottlenecks imposed by the transfer between different portions of the antenna (often referred to as “compartments”) might be present. This constitutes a diffusion limitation to the overall trapping kinetics, which is of a different nature with respect to the pure antenna size affect (see below).

Descriptions of the excited state kinetics both in the core of PSII as well as in the PSII-LHCII supercomplex (e.g., [201, 211, 212, 215]) based on kinetic models dominated by limitations imposed by photochemical trapping have been presented. Although a limitation imposed by excited state diffusion was observed in these studies, it was concluded that this process plays a minor role in determining the effective trapping rate with respect to the kinetic bottleneck at the level of charge separation events [201, 209, 211]. On the other hand considerable evidence has also accumulated indicating that there is a partial but significant diffusion limited component in PSII, which accounts for 20-30% of the overall trapping time. We underline that this excited state diffusion limitation to photochemical trapping kinetics (associated to the antenna) is not the "antenna size" effect that is dealt with below, rather, it is due to kinetic "bottlenecks" in the energy transfer either between different antenna complexes or between specific pigments in these complexes [198, 210, 224, 225]. Selective quenching of the fluorescence of core chlorophyll-protein complexes by photochemistry indicates that PSII is partly diffusion limited. In this context it is interesting to note that *in vivo*, in the presence of loosely bound L-LHCII, a clear slowdown of the PSII kinetics is evident as compared with purified PSII-LHCII supercomplexes, which contain only S- and M-LHCII [62, 216]. This implies that excitation energy transfer from L-LHCII has a rate limiting effect on overall PSII trapping time and could represent the kinetic "bottleneck" originally discussed by [198].

A pronounced diffusion limited component, estimated to be just above 50% of the overall trapping time, has also been demonstrated in PSI [116, 150]. In this case the kinetic "bottleneck" is associated with the uphill energy transfer from the red forms to the reaction centre. Thus, of the two photosystems, PSI would appear to have a greater contribution by the kinetics of energy diffusion within the antenna to the photochemical trapping kinetics.

One should distinguish between the "diffusion limited" concept imposed by kinetic bottlenecks, briefly discussed above, and the limitations associated to the "antenna size", which is certainly very important in determining the overall trapping kinetics. It has been suggested for many years that the overall trapping rate should scale with the antenna size, on the basis of simple theoretical considerations [75, 189-192, 226], though it has not been experimentally demonstrated until fairly recently. Analysis of a range of PSII-LHCII supercomplexes harbouring antenna of progressively increasing sizes, purified by mild detergent treatment of purified grana membranes [62], showed that the average excited state migration time did indeed show an almost linear dependence to the dimension of chromophore array, thus respecting, to good approximation, the theoretical predictions. Similar conclusions were previously reached by the analysis the of PSII core complexes and the PSII-LHCII supercomplex embedded in the thylakoid membrane, and thus closer to the natural environment, by comparing wild type and antenna lacking mutants of Barley [196].

It is also worth mentioning that in the analysis of PSII-LHCII complexes with different antenna sizes, both isolated [62] as well as in the thylakoid membranes [196], in order to explain the experimental results it was necessary to consider also a small difference of the photochemical trapping rate. This is probably a secondary effect linked to removal of the antenna and becomes significant for particles with smaller antenna size. The proposed slower photochemical trapping resulting from the systematic removal of the external antenna is an interesting observation which still requires further experimental investigation in order to be fully understood.

Excited state diffusion limitation due to the "antenna size" effect are more difficult to demonstrate for PSI. This is because it is more challenging or impossible to prepare supercomplexes with progressively increasing antenna sizes, as in the case of PSII, and because the interpretation of the experimental results is complicated by the presence of low energy Chl forms that do not permit utilisation of the approximation of an isoenergetic antenna. Nevertheless, it has been possible to compare the excited state relaxation kinetics of PSI core complexes [197, 204], PSI(-LHCI) [116, 162, 195, 197, 204, 206, 227], PSI-LHCII supercomplexes [48, 150], as well as of PSI complexes with a reduced (about halved) LHCI complement [228]. In all of these studies, it was shown that the excited state decay relaxation is markedly faster in the core complex of PSI compared to that of supercomplexes harbouring different antenna complements. However, the removal of the LHCI antenna not only decreases the dimension of the antenna, but also leads to removal of low energy forms from the antenna, so that the effect of these two processes cannot be simply distinguished. Only in the PSI-LHCII supercomplex [48] the antenna size was varied, in this case increased, without altering the red form content in the system.

A very clear manifestation of the impact of the low energy spectral forms in PSI on the kinetics of excited state relaxation, which can be investigated by fluorescence lifetime analysis, is that whereas in PSII, which can be considered as an almost isoenergetic system, the kinetics are substantially independent on the wavelength at which they are monitored, in the case of the PSI supercomplexes there is a gradient of (average) lifetime values. These values range from 20-40 ps for short wavelength emissions, dominated by contribution from the core and “bulk” (non red-shifted) forms in the antenna to values of 60-100 ps for the red tail of the emission, dominated by the red forms [48, 116, 197, 199], which is a remarkable difference. The average lifetime ($\tau_{\mathrm{av}}$) can be described to a good approximation by a linear combination of an average excited state migration time ${\bar{\tau }}_{\mathrm{mig}}$ and an average trapping time ${\bar{\tau }}_{\mathrm{trap}}$, according to the relation $\tau_{\text{av}}^{-1}={\bar{\tau }}_{\text{mig}}^{-1}+{\bar{\tau }}_{\text{trap}}^{-1}$ [190]. Since the value of ${\bar{\tau }}_{\mathrm{trap}}$ describes a molecular process, the photochemical charge separation, which is meant to be independent of the observation wavelength, the large variation of $\tau_{\mathrm{av}}$ as a function of wavelength must reside in a gradient of values of ${\bar{\tau }}_{\mathrm{mig}}$, the contribution of which increases as the low energy spectral forms becomes more red-shifted. This, as mentioned above, is a clear manifestation of a diffusion-limited situation imposed by a kinetic bottleneck rather a limitation due to the size of the antenna. Such an interpretation is also confirmed by the observation that the wavelength dependence of $\tau_{\mathrm{av}}$ is substantially lost in the PSI core isolated from plants, in which low energy forms are substantially absent [139, 197]. Another evidence of the impact of the red forms in determining the excited state lifetimes comes from the analysis of core complexes of PSI isolated from different cyanobacteria strains [50, 223, 226, 229]. Differently from higher plants, low energy spectral forms are located in the core complex of these organisms. Moreover, different species harbour red forms absorbing and emitting at different wavelengths in the 710-750 nm range [50, 229]. Thus, by comparing core complexes of PSI isolated from different cyanobacteria, it is possible to compare systems with analogous, if not identical, antenna size, but with a different energy spread. Such comparative analysis indicated that, also in this case, the kinetics of excited state relaxation become progressively slower as the spectral shift of the red forms increases [50, 229], which is in good qualitative agreement with the results obtained in higher plant systems. Although a variation of average lifetimes through the emission band is observed in all the mentioned studies [50, 116, 162, 195, 197, 204, 227, 229], it is more or less pronounced depending on the spectroscopic properties of the complexes, in particular on the abundance of the red forms. For instance, it has been argued by [204] that the most relevant process in determining the excited state kinetics is photochemical trapping rather than excited state equilibration. Yet the PSI complex they analysed appears to have a lower red form content compared to that studied by others [116, 195, 197, 199] so that the influence of the red form might be less relevant. In any case, rather than the presence of such phenomenon, it is the extent to which it contributes to overall excited state kinetics that is still somewhat debated.

### 3.2. Photochemical Energy Conversion and Charge Separation Reactions

Conversion of the absorbed photon energy into electrochemical potential, which is ultimately used by the cell metabolism, represents the central catalytic reaction of the photosynthetic process. Photochemical charge separation can be described, in general terms, as the reaction through which the lowest singlet excited state residing on the chromophores composing the reaction centre (RC*) is converted into a radical pair of the form [D^+^A^–^], where D^(+)^ is the (primary) electron donor and A^(–)^ is the (primary) electron acceptor. The radical pair formed by the primary photochemical reaction is stabilised through a series of electron transfer (ET) steps involving the other redox-active cofactors bound to the reaction centre subunits (Fig. **1C**).

Although both photosystems share a common general principle of primary photochemistry and a rather similar structural organisation of the chromophores involved, there are also specific and often significant differences in the reaction mechanism between PSI and PSII. In the following paragraph we will discuss aspects that can be considered common to both reaction centres and those in which they differ markedly. The most obvious difference which has emerged in the past decade is that in PSII electron transfer and photochemistry involve only one of the two symmetry related cofactor chains bound to the reaction centre subunits (asymmetric or monodirectional electron transfer, for reviews see [41, 230]), whereas in PSI both branches are active (symmetric or bidirectional ET) (for reviews see e.g., [44, 45, 231-233]) also at the level of primary charge separation [43]. Thus, when comparing the kinetic properties of both reaction centres, it is necessary to bear in mind differences, in particular since a large body of literature about primary charge separation has been published before solid evidence for bi-directionality in PSI became available. It is possible to compare the properties of the two photosystems by an approximation in which the events occurring on two active branches of PSI are considered as an “overall” process that can be reduced to the monodirectional case.

As described above, the photochemical reaction centre of both photosystems is composed of 6 chlorine pigments: in the case of PSI, these are six Chls *a*, one of which is 13’ epimer [17, 18, 234], whereas in PSII four are Chls *a* and two are Pheo (Fig. **1C**). The pigments are more tightly packed in the case of PSI, with average molecular centre-to-centre distance of the order of 4 Å [20, 235] that increases to 8 Å in the case of PSII. This means that interactions amongst pigments comprising the reaction centre of PSII are, in general terms, weaker than those of PSI RC pigments. Another very clear difference which emerges from the comparison of crystallographic models of PSI and PSII is the orientation of the A_0 _(PSI) and Pheo (PSII) molecules with respect to the C_2_ symmetry axis: the plane of both Chls A_0_ is almost parallel to the eC2 Chls, i.e., forming an angle of ~40° with respect to the symmetry axis, whereas the plane of both Pheo is almost parallel to the axis and not far from perpendicular to that of the accessory chlorophylls Chl_D1/2_ (Fig. **1C**). Inspection of the PSII core high resolution structures obtained in cyanobacteria [20], which probably can be considered as an acceptable model also for the higher plant core, suggests that the porphyrin plane of all molecules bound to the PSII reaction centre subunits are more distorted with respect to those bound to PSI, which appear to overlay with a reference plane passing through the nitrogen atoms in the ring. The distortion of the Chl plane in PSII might be more evident because of the higher resolution of this structural model (1.9 Å) [20] compared to that both of bacterial and higher plants PSI (2.5-3.3 Å) [17, 18]. Yet, chlorophyll deformations were observed and analysed in the structural model of LHCII [122], which has a resolution similar to that of the PSI core isolated from *S. elongatus *[18]. Since it is possible to correlate the deformation of the Chls with the modulation of frontier orbital, which determine both the optical [180, 236, 237] and redox properties of the bound cofactors, this observation suggests that PSI and PSII have evolved different strategies to tune the properties of the cofactors involved in primary photochemical reactions. Yet, for both PSI and PSII, a pair of Chls that form a face-to-face dimer at the interface of the two main RC subunits is found. Though there are differences in their centre-to-centre distances and the overlap of Chl ring planes, they are identified with the so-called “special (Chl) pair” and named P_680_, in PSII and P_700_ in PSI. These are the first cofactors discovered to be functionally involved in primary electron transfer reactions. There are some clear differences between the “special pairs” of PSI and PSII: i) the absorption spectrum is red shifted in PSI [238-240], showing a bleaching upon oxidation at ~700 nm (P_700_) compared to that of P_680_ (~685 in intact systems) [241-243]; ii) when comparing the P^+^–P (or ^3^P–P) difference spectra, a very resolved spectral structure is observed in the case of P_700 _showing features at ~ 685 and 675 nm [244-247], whereas almost a single bleaching peaking at 682-685 nm, with a shoulder near 680 nm, is observed for the case of P_680_^+^–P_680_ difference spectrum [241, 248-250]; these differences are likely to arise from the stronger interaction with the neighbouring pigments in the case of PSI reaction centre pigments compared to those comprising the PSII RC; iii) there is a the large difference in redox potential of the P_700_^+^/P_700_ (~500 mV) [251, 252] with respect to the P_680_^+^/P_680_ (~1.2 V) [41, 230, 253] redox couples, which is required for the different catalytic activity of the two reactions centres (Table **1**); iv) finally, the cation species have markedly different lifetimes. P_700_^+^ is reduced by plastocyanin with kinetics which are of the order of 6-60 μs [254-256] and has a lifetime of the order of several tens of ms in isolated PSI complexes in the absence of an exogenous donor; P_680_^+^ is characterised by a lifetime of the order of 20-40 ns [254, 257-260], as it is rapidly reduced by Tyr_Z_ that acts as in intermediate in electron transfer between the OEC and P_680_^+^.

It has been generally considered that both [P_680_] and [P_700_] acted as the primary electron donor reducing directly the respective (primary) electron acceptors Pheo_D1_ and A_0 _and hence forming the radical pairs [P_680_^+^Pheo^–^] and [P_700_^+^A_0_^–^]. These reactions were, at least initially, considered to be very slowly reversible in terms both of RC direct repopulation (RC*) and recombination to the neutral (ground) state, also because of coupling by rapid oxidation of the primary acceptors by the next cofactor in the redox chain, plastoquinone Q_A_ and phylloquinone A_1_ in PSII and PSI, respectively. Although the detailed molecular mechanism of charge separation are still debated in both RCs of higher plants, particularly the details concerning the precise rate of the reactions and the energetic factors which determine and control these rates, there is a substantial agreement in considering the population of the states [P_680_^+^Pheo^–^] and [P_700_^+^A_0_^–^] as the first “stable” radical pairs. Thus, in this framework, the photochemical population of [P_680_^+^Pheo^–^] and [P_700_^+^A_0_^–^] could be considered equivalent to the “trapping” of the excitation energy into a chemical form.

The first experimental evidence that questioned the model of an irreversible population of radical pair states on the time scale of the excited state came from the analysis of the fluorescence lifetime decay in PSII [215]. It was suggested that radical pair is actually (rapidly) reversible leading to repopulation of the RC* and ultimately of the pigments in the antenna system. This is known as the reversible radical pair (RRP) model for primary charge separation. Even though the rate constant for primary charge separation population and that of the reverse reaction, hence the equilibrium constant and standard free energy difference associated with it, are somewhat debated [62, 210, 211, 214, 215, 219], rapid reversibility of primary photochemistry in PSII is accepted to be a relevant factor in determining the effective trapping kinetics. More recently, it has also been proposed that rapid reversibility of primary charge separation is also a feature of the molecular mechanism of energy conversion in PSI [42-44] and was proposed to take place both considering a monodirectional [42] as well as a bidirectional [43, 44] ET model. It is interesting to compare the values associated with the primary charge separation and the equilibrium constant for PSII and PSI (as a collective rate comprising both ET branches), even considering that the spread of values present in the literature is large. For PSI, in which the number of reports concerning RRP is limited, the values of the charge separation rate constants from the lowest single excited state of the equilibrated RC vary within the rather narrow range of 200-400 ns^-1^ [42-44, 261] and the associated equilibrium constants (*K_eq_*) are in the range of 10-20. For the case of PSII the spread of rate values is greater, ranging from 400-10 ns^-1^ [129, 196, 209-211, 214-216] and *K_eq_* in the range 4-90 in the above mentioned studies. The exact values depend on the kinetic model used to describe the experimental results, and that is one of the reasons for such variations since all the different modelling approaches contain different levels of approximation. Nevertheless, one apparent difference is that rate constant values reported for PSI are generally larger, i.e., the reaction is faster, compared to that of PSII. This is in agreement with the very weak fluorescence emission of PSI at room temperature.

**Fig. (6):**
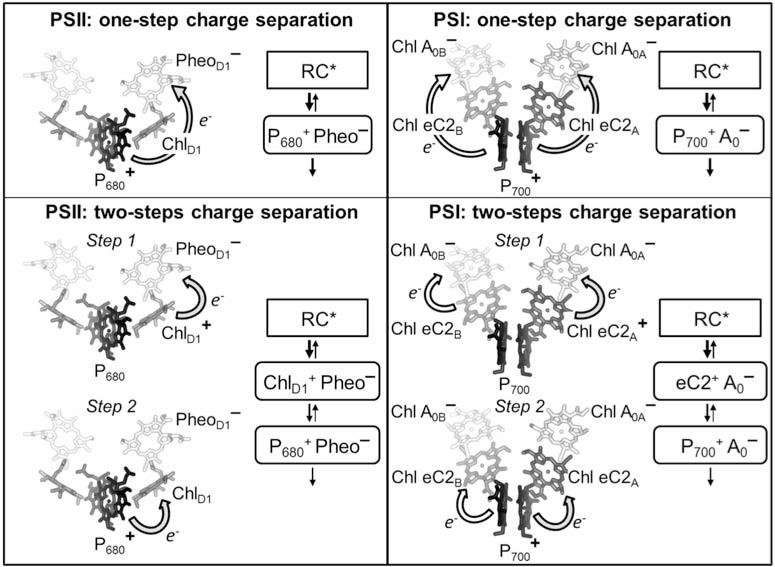
Sequence of electron transfer events for the monidirectional mechanism of PSII (left) and the bidirectional mechanism of PSI (right). PSII: One-step charge separation: the radical pair (P_680_^+ ^Pheo_D1_^115%; 'Times New Roman',serif">–^115%; 'Times New Roman',serif">) is populated directly, the accessory chlorophylls do not play a direct role in ET reactions; Two-steps charge separation model: the radical pair (P_680_^+ ^Pheo_D1_^115%; 'Times New Roman',serif">–^115%; 'Times New Roman',serif">) is populated by two sequential events, the first of which is more likely the oxidation of the accessory Chl_D1_ and concomitant reduction of Pheo_D1_. The second step in the ET chain is then the reduction of Chl_D1_^+^ and concomitant oxidation of P_680_. PSI: One-step charge separation: the radical pair (P_700_^+ ^Chl A_0_^115%; 'Times New Roman',serif">–^115%; 'Times New Roman',serif">) is populated directly, the accessory chlorophylls do not play a direct role in ET reactions; Two-steps charge separation model: the radical pair (P_700_^+ ^Chl A_0_^115%; 'Times New Roman',serif">–^115%; 'Times New Roman',serif">) is populated by two sequentioal events, the first of which is more likely the oxidation of the accessory Chl eC2 and concomitant reduction of Chl A_0_. The second step in the ET chain is then the reduction of Chl eC2^+^ and concomitant oxidation of P_700_. Note the presence of two functional ET chain in the case of PSI.

So far we have discussed the case in which charge separation leading to formation of [P_680_^+^Pheo^–^] and [P_700_^+^A_0_^–^] occurs in a single, more or less rapidly reversible, step. In the classical model, charge separation stems from the special pair leading to the direct reduction of the acceptors Pheo^–^ and A_0_^–^, without direct involvement of the accessory Chls (Fig. **6**). Given the structural organisation of the cofactors, this is unlikely. The accessory chlorophylls Chl_D1_ in PSII and eC2_A/B_ in PSI should then either be part of “primary donor”, as a sort of extended dimer or cluster of pigments, or part of the “primary acceptors”, forming a sort of dimer. This scenario is not impossible due to the almost face-to-face configuration of eC2_A/B_ and A_0(A/B)_ in PSI. It has been actually suggested that A_0_ might be a Chl dimer [44, 194, 262, 263]. However, in the case of PSII, Chl_D1_ and Pheo are arranged nearly perpendicular to each other so that strong interactions between these pigments are damped for geometrical reasons. Thus, the accessory Chl_D1_ is expected to play a direct role as an electron transfer intermediate. Thus, the [P^+^_680_Pheo^–^] is formed by a two-step charge separation mechanism (Fig. **6**). Indeed, there are several suggestion in the literatures, which were initially based on mutagenesis analysis of residues involved in the binding of RC pigments [40] and later substantiated by a host of reports based on kinetic analysis of fluorescence decay [62, 209-211, 216, 221, 264] as well as ultrafast transient absorption data [201, 218, 265], that suggest that charge separation in PSII is better described by considering a minimum of two consecutive radical pairs. Thus the accessory Chl_D1_, rather than P_680_ would be the primary donor. In compact form, the reaction scheme could be summarised as RC*↔[Chl_D1_^+^Pheo^]–^→[P_680_^+^Pheo^–^]→ (see Fig. **6**).

Rapid reversibility of the secondary radical pair (P_680_^+^Pheo^–^↔Chl_D1_^+^Pheo^–^) has also been suggested [201, 209-211, 264]. However, in general terms, the value of equilibrium constant for this reaction appears to be about half that of primary charge separation. Moreover, the actual rate constant of the second step in the charge separation reaction is reported to be about two to ten times slower. Most common values to describe the successive reaction leading to oxidation of Pheo^–^ and reduction of Q_A_, are in the range of 2-10 ns^-1^ (e.g., 62, 201, 209-211, 215, 264) and is substantially irreversible.

Evidence for a direct involvement of the accessory chlorophyll in the charge separation reaction has also been presented for the case of PSI in recent years [42, 43]. This was based on evidence that: a two-step charge separation mechanism provides a better description of the kinetics on an ultrafast time scale [42]; considering the accessory Chls eC2_A/B_ as the primary donor provides a better description of the transient absorption spectra as well as of the effect of mutations in which the coordination of A_0(A/B)_ was modified [43]. This would yield a general reaction scheme of the kind just discussed for PSII, being RC*↔[eC2^+^A_0_]^–^[→P_700_^+^A_0_^–^]→, with the difference that, in the case of PSI, charge separation statistically takes place on two parallel chains (A and B branches, Fig. **1C**, Fig. **6**). Similar to the case of PSII, the value of the rate constant associated with the second step in the reaction mechanism has been suggested to be about a half that of the primary photochemical separation, i.e., around 70 ns^-1^, but slowly reversible due to a large equilibrium constant greater than 100 [43]. The oxidation of A_0_^–^ by the successive acceptors A_1_ appears to be described by a rate constant of the order of 20-40 ns^-1^ [44, 50, 203] which is instead about ten time faster compared to the values commonly reported for Pheo^–^ oxidation. In terms of the rate constant for a given ET transfer reaction this seems to be the largest difference amongst cofactors bound to the two RCs.

Finally we turn to consider the different maximum quantum photochemical efficiency ($\phi_{\mathrm{pc}}$) of the two photosystems, i.e., the ratio of the number of converted and absorbed photons when the RC centre are open (fully oxidised acceptors). From a mathematical point of view this parameter can be expressed as $\phi_{\text{pc}}^{}=k_{pc}^{}/({\sum k}_{d}+k_{pc}^{})$, where $\sum k_{d}$ represent the sum of the rate constants associated with all non-photochemical excited state deactivation processes (fluorescence, heat, intersystem crossing, etc.) and *k*_pc_ is a macroscopic constant which describes excited state trapping, including contribution from excited state equilibration. *k*_pc_ can be demonstrated to be related to the experimental value of the average lifetime $\tau_{\mathrm{av}}$ by the relation $k_{\text{pc}}^{-1}\sim \tau_{av}\equiv \sum_{i}^{}f_{i}\tau_{i}$ [199], where are the different lifetimes and $f_{i}$ are the fractional amplitudes associated to the description with a sum of exponential functions of the fluorescence decay, which is the most common experimental approach to determine such quantities. Based on measured lifetimes, the calculated values of $\tau_{\mathrm{av}}$ for PSI are in the range of 20-100 ps and considering a value of $\sum k_{d}$ equal to (1.5-3) ns^-1^, which is an average value for the decay of fluorescence in isolated antenna complexes and hence in absence of photochemistry, one calculates values of *ϕ*_pc,PSI_ larger than 0.95 with limits up to 0.99 [48]. On the other hand in PSII the value of is of the order of 150-350 ps, respectively in isolated PSII-LHCII supercomplexes [62] or in thylakoid membranes where additional loosely bound LHCII trimer are present [216]. This implies that the maximum *ϕ*_pc,PSII_ value is comprised between 0.8 and 0.9, which compares well with the estimates obtained by fluorescence induction methods [67]. The larger photochemical efficiency of PSI is related to the more rapid overall trapping kinetics, which might be due to the bidirectional charge separation mechanism. Actually, considering an average value for the overall rate associated with primary photochemistry for PSI, i.e., ~200 ns^-1^ and considering the presence of two equal branches (slight differences have been reported but this will not affect our reasoning), this would correspond to a rate of about ~100 ns^-1^ on each branch which falls about the mean values of the spread considered for PSII reactions. Bidirectional charge separation can simply be envisaged as a way to increase the probability of de-excitation of RC* photochemically rather than by non-productive trivial decay, directly or by repopulation of the antenna excited state. The indication that formation of the secondary radical pair is substantially irreversible in the case of PSI, whereas it is considered to be partially reversible in PSII, might, despite the broad spread of values present in the literature for such parameters, also play a role in ensuring the outstanding quantum efficiency of PSI.

## 4. PHOTOINHIBITION, REGULATION OF LIGHT HARVESTING CAPACITY AND PHOTOPROTECTIVE STRATEGIES

### 4.1. Photoinhibition

Typical turnover time for the linear electron transport in thylakoid membranes are of the order of a few milliseconds, the rate limiting steps being the dissociation of fully reduced and protonated Q_B_ from its binding site in PSII, the binding of an oxidised PQ molecule at the Q_B_ site and oxidation of PQ during the turnover of Cyt *b*_6_*f* [254]. Hence, in their natural environment plants can frequently experience photon flux densities that exceed the saturation of whole linear electron transfer chain. Under such circumstance the light absorbed by the photosystems is not efficiently converted as it is in part dissipated both via radiative and non radiative de-excitation processes of the singlet excited state. This does not only lower the photochemical yield under steady state conditions with respect to its maximal value attained at limiting light regimes, but it can also lead to damage to the photosynthetic apparatus, a process which is known, overall, as photoinhibition. The rate of the light-induced loss of photochemical activity, particularly that of PSII, which appears to be the principal target of photoinhibition, was demonstrated to display a linear dose-effect relationship both in isolated thylakoids [266-269] as well as in leaves treated with protein synthesis inhibitors [270]. However, *in vivo*, photoinhibition becomes significant only when the rate of PSII damage exceeds the rate of repair, which is determined by the PSII RC protein turnover (reviewed by [35, 63, 271]). Although the basal turnover rates both of D2 and in particular of D1 are high, it was also shown to increase when the organisms are exposed to saturating light regimes [35, 63, 271-275].

Photoinhibition is a complex process that can be caused by different molecular mechanisms, involving both the donor side and the acceptors side of the reaction centre, as well as mechanisms that involve excited states of the antenna pigments. We begin by discussing this latter process. The average excited state lifetime of PSII *in vivo* is known to increase from approximately 350 ps (see “excitation energy transfer” section) when the centre is photochemically active (“open”, *F_0_*), to about ~2 ns, when the acceptor Q_A_ is reduced and the centres are “closed” (*F_M_*). This corresponds to about a five/six fold increase of PSII fluorescence emission under steady state conditions. In the absence of photochemical quenching, the Chl excited state decays by its “natural” deactivation pathways, which include other than fluorescence and heat, the population of the excited triplet state via the intersystem crossing mechanism. In solvated Chl, the yield of intersystem crossing (which has a rate constant of about (8 ns)^-1^) is about twice that of fluorescence corresponding to a yield of approximately 0.6 [276]. The relative yields of fluorescence emission and ^3^Chl* population for pigments bound to photosynthetic complexes have been estimated to be the same *in vivo* as in organic solvent [277] and the actual values *in vivo* (when photochemistry is absent) are not much lower than those in solvent [278, 279]. The energy of the ^3^Chl* is sufficiently high (~1.3 eV) and the lifetime of this state is sufficiently long (1-3 ms), permitting interaction with the triplet ground state of molecular oxygen [280]. This interaction leads to collisional quenching of the pigment triplet excited state and population of the excited oxygen singlet state (~1.0 eV), which is a highly reactive species. Since the excited Chl triplet state is populated from its singlet excited state, the increase in the excited state lifetime when the RC are closed, i.e., at *F_M_* and in any situation of continuous illumination for which the emission is higher than *F_0_*, leads to a progressive increase in the population of ^3^Chl* which is potentially harmful. Yet, even in place of a high Chl triplet formation, its population is kept at extremely low level by efficient triplet-triplet energy transfer to complex-bound carotenoids [277, 281-283]). The energy of excited carotenoid triplet state (^3^Car*) is lower than that of ^3^Chl* [284-286] and its lifetime is shorter, 3-7 µs (reviewed by [284-287]), so that interaction with molecular oxygen is prevented. Indeed the presence of Chl triplet states (populated by intersystem crossing) in relatively intact systems such as isolated thylakoid membranes has been detected only recently [288-290] and only at very low temperatures (~2 K), whereas the ^3^Car* are commonly observed, both in intact systems [277, 281-283, 291, 292] as well as in the isolated core [293-295] and antenna complexes [296-302]. Recently the presence of ^3^Car* has been also observed in PSI embedded in thylakoids [292] as well as in isolated Lhca [303, 304], suggesting that ^3^Chl* quenching by carotenoids is a general process occurring in photosynthetic Chl-protein complexes. Differently from the case of Chl, the population of ^3^Car* by intersystem crossing occurs with a very low yield, due to the extremely short excited state lifetime of ^1^Car* (1-3 ps). The population of ^3^Car* by ^3^Chl* sensitisation is not only the principal molecular mechanism by which this state is formed, but it also represents a crucial strategy of protecting the system from photo-oxidative damage. This is also demonstrated by the observation that both in organisms treated with inhibitors [305, 306] as well as in mutants impaired at early stages of carotenoid biosynthesis [307-311], when subjected to intense oxidative stress, rapid bleaching of pigmentation occurs and lethal phenotypes at early stages of greening are produced. Interestingly mutants that lack almost completely long chain carotenoids are also incapable of assembling the external antenna of both photosystems as well as the core subunits of PSII [311], indicating that these molecules have also an important role in stabilising the protein ternary structure (see Fig. **1B** to appreciate the large amount of Cars in photosystems, especially in the Lhc complexes). Similar conclusions were obtained by the analysis of *in vitro* reconstituted antenna complexes by changing the carotenoid content during protein refolding [312].

Nevertheless, photoinhibition caused by singlet oxygen sensitisation by ^3^Chl* has been suggested to play a significant role in high light stress induced by visible radiation, based on detailed action spectra determined as loss of maximal PSII quantum efficiency [269, 313-315]. The action spectra were interpreted in terms of a small, heterogeneous, population of Chl-binding complexes in which Chl-Chl energy coupling is substantially eliminated and thus Chl-Car interactions are perturbed, so that ^3^Chl* quenching is inefficient [313]. It is reasonable to correlate such a population of complexes with the ^3^Chl* states associated with PSII that have been detected in isolated thylakoids at low temperature [288, 290].

Several other mechanisms that can lead to photooxidative damage, particularly at the level of PSII, have been discussed. In general they could be classified as “acceptor side” and “donor side” photoinibition, depending on which ET cofactors are either involved or represent the target of photoinhibition (extensively reviewed by [271, 316-319]).

The acceptor side photoinibition occurs when the plastoquinone pool is highly reduced, i.e., under conditions that saturate the thylakoid transport chain and the Q_B_ binding pocket in PSII remains unoccupied due to the limited availability of oxidized plastoquinones molecules. Since PQ diffusion is a relatively slow process (up to 10 ms for purely diffusion limited occupancy of Q_B_ site) [320], this leads to a stabilization of Q_A_^–^ that in extreme reducing conditions can be doubly reduced and protonated to the quinol form and leave its binding site [321, 322]. The absence of an electron acceptor at the photosystem acceptor side promotes the formation of the excited triplet state of P_680_ (^3^P_680_*) via charge recombination of the precursor radical pair (which is initially formed in a pure singlet state), according to the scheme: ^1^[P_680_^+^Pheo^–^]↔^3^[P_680_^+^Pheo^–^]→^3^P_680_Pheo [323-326]. This is due to the lengthening of ^1^[P_680_^+^Pheo^–^] lifetime (to ~20 ns) when oxidation of Pheo^–^ is blocked in the absence of Q_A_ [243], allowing enough time for the (singlet) radical pair to diphase to its triplet state. ^3^P_680_*, like other Chl triplet states, reacts with molecular oxygen to produce singlet oxygen that can easily destroy proteins and pigments of PSII [243, 319, 327, 328]. An interesting observation is that ^3^P_680_* is populated with a high yield only when Q_A_ is either fully reduced or its binding site is vacant [250, 288, 313, 323-325, 329]. In this case the lifetime of the Chl triplet formed by charge recombination in PSII (^3^P_680_*) is in the order of 1 ms, which is a typical value of the Chl triplet state [288, 289, 330, 331]. However, when Q_A_^–^ is present in the binding site, the long-lived ^3^P_680_* is not observed [288, 313, 330, 331]. Instead, a triplet state that is also likely associated with the reaction centre pigments and populated by the recombination mechanism [289, 331] and that has a ~20 μs decay time [288, 313, 330, 331] has been detected. The quantum yield of the rapidly decaying RC triplet appears to be smaller than that of ^3^P_680_* observed under reducing condition, and comparable with that of “antenna” ISC-populated Chl triplets. Although the mechanism by which the presence of Q_A_^–^ affects the yield of charge recombination is not fully understood, it could be considered as an “intrinsic” protective strategy that acts by reducing the yield and the lifetime of the RC recombination triplet. Moreover, the reducing conditions necessary to observe the long-lived ^3^P_680_* *in vitro* (typically poising the ambient redox potential at about –500 mV) are probably seldom encountered in physiological conditions. Another observation which has received little attention is that, at least at low temperatures, the recombination triplet yield in PSI (^3^P_700_*) under non reducing conditions, appears to be higher with respect to that of PSII [288]. Yet, it has been shown that ^3^P_700_* is not quenched by oxygen [332, 333] and should therefore have minor contribution to photooxidative stress.

Photoinhibition of PSII might also occur because of impairment of the photosystem donor side. This inhibitory process typically occurs when the reduction of the P_680_ radical cation (P_680_^+^) from the oxygen-evolving complex is interrupted. Since the standard redox potential of P_680_^+^ is estimated at approximately 1.2-1.3 V, making it one of the most oxidising species in nature, in the absence of rapid reduction by Tyr_Z_/OEC, it can oxidize other amino acid residues, not involved in the electron transfer chain, leading to the damage of PSII reaction centre proteins [334, 335]. Indeed in systems in which the OEC is impaired or removed by biochemical treatments, such as Tris washing, the yield of photoinhibition increases by several orders of magnitude [336]. Under physiological conditions it has been proposed that the population of the photo-induced excited state of some S-states which are part of the OEC turnover, might represent the principal source of photo-oxidative damage both by visible as well as ultraviolet radiation [318, 337]. At the same time, under conditions that promote a relatively long lived P_680_^+^, alternative electron transfer chains, involving one of the β-carotene, one of the distal Chls bound to D1-D2 (Chl_Z_, Fig. **1B**) and possibly Cyt *b*_559_, have been observed within the PSII reaction centre [327, 338-342]. Those might be seen as “safety valves” which can sustain a low yield of electron transfer in PSII when the donor side is impaired and, by reducing P_680_^+^, lowering the yield of oxidative damage.

Although PSII is the main target of photoinibition, presumably due to its longer excited state lifetime with respect to PSI, also PSI can be photodamaged under stress conditions that impair the thylakoid electron flow [343, 344], in particular when there is a strong limitation of the PSI diffusible electron acceptor pool. This is the case for instance at low temperature [345, 346], which slows down the activity of Calvin cycle enzymes and thus NADPH is not utilised and reduced ferredoxin is accumulated. The mechanism of PSI photoinibition is mainly associated with the oxidative destruction of the electron carriers (F_X_, F_A_ and F_B_) on the acceptor site of PSI by oxygen radicals, mainly superoxide (O_2_^–^) [346], which is produced by the Mehler reaction, probably at the level of reduced F_A/B_. In a similar way as in PSII, photoinibition of PSI causes the degradation of the reaction center subunits PsaA and PsaB [347]. However the mechanism of PSI repair is very different compared with PSII, where mainly the D1 subunit is subjected to a fast turnover [35, 275, 348, 349], while the remaining subunits are mostly re-used during the reassembly process [36, 63, 348]. In the case of PSI, all the PSI core subunits are degraded and apparently none of the core subunits is re-used [350]. Only Lhca subunits seem to be utilised several times during the PSI assembly process, as it was observed that their turnover is slower compared to that of PSI core subunits [350]. The “modular” architecture of PSII core subunits, compared with the more “monolithic” organisation of the PSI core, appears to serve at the level of the rapid turnover of the PSII reaction centre, due to its higher sensitivity to photoinhibition. However a different report suggests that the Lhca antenna complexes are destroyed faster than the core complex and this result has been interpreted as Lhca acting as a safety valve to protect PSI core [351]. A higher yield of photo-oxidation of Lhca subunits appears consistent with the observation that most of the singlet excited states in PSI reside on the red forms bound to the Lhca3 and Lhca4 subunits at room temperature [160] and in turn the Chl triplet state should be principally populated in this pigment pool. At the same time, the red forms were shown to be efficiently coupled to carotenes, leading to population of ^3^Car* that should prevent singlet oxygen production [292, 303]. This proposed mechanism of protection appears to be different from PSII, where Lhcb antennas switch to a “dissipative” mode to lower the singlet excited state population of PSII through the so-called non-photochemical quenching (NPQ) process that will be discussed in further detail below. At the same time, the excited state lifetime of PSI remains very low (~ 40 ps) even when P_700_ is oxidised (it has been proposed that P_700_^+^ is an efficient ^1^Chl* quencher), often significantly below that of PSII under maximum quenching exerted by NPQ. Further investigations are required to elucidate this issue.

Other mechanisms to reduce oxidative sensitised photoinibition of PSI are the induction of enzymes involved in scavenging of ROS (superoxide dismutase, SOD; ascorbate peroxidase, APX) and, probably, cyclic electron transfer around this photosystem. The reduction of molecular oxygen to the superoxide radicals (O_2_^–^) has been observed at the acceptor side of PSI, and it has also been suggested to have a physiological role in the transition from a dark adapted condition to normal operation of the linear flow under steady state illumination [352]. Superoxide anion radicals are disproportionated by SOD in hydrogen peroxide (H_2_O_2_) that is consequently reduced to water by APX which uses the ascorbate (vitamin C) as a redox cofactor [353, 354]. This is known as the water-water cycle because the origin of electrons is the water split by PSII [355]. During stress conditions the electron flow to molecular oxygen is increased due to an unbalanced production/consumption of reducing power, which causes accumulation of NADPH. Therefore an efficient water-water cycle avoids the interaction of O_2_^–^ with the surrounding proteins and their damage.

### 4.2. Regulation of Light Harvesting Capacity and Photoprotective Strategies

The photosynthetic process in plants is regulated by different mechanisms that influence either the efficiency of light harvesting or the balancing of the absorption cross section of the two photosystems in response to different light intensities and spectral qualities. Some of these mechanisms involve the synthesis of new proteins, in order to adjust the Lhc to core complexes ratio or the change the PSII to PSI ratio. These are relatively long term mechanisms [106, 154, 356-358], which will not be discussed further. On the other hand, other mechanisms respond rather promptly (from seconds to tens of minutes) to changes in the environmental light conditions. The most intensively investigated are: i) non photochemical quenching (NPQ) of Chl fluorescence, which acts at the level of PSII [10, 359-361], although NPQ at the level of PSI has been proposed [362]; NPQ regulates the efficiency of light harvesting, especially under light intensities that exceed or are about at the level of saturation of the thylakoid electron transfer chain, by increasing the dissipation of Chl singlet excited state by heat; ii) State I-State II transitions, or simply State Transitions (ST), which involve the reversible migration of a fraction of LHCII from PSII, to which they are associated in State I, to PSI under light conditions in which the absorption rate of the two photosystems is unbalanced in favour of the former (State II) [363] (for extensive reviews see [88, 364, 365]). As both processes are observed as a decrease in the maximal fluorescence emission level at the steady state, it is not always simple to distinguish between NPQ and ST, and often ST are considered as a “component” of NPQ. However they differ significantly in their nature, i.e., ST are not strictly speaking a quenching process, rather they are a change in the optical cross section of PSII by LHCII transfer to the weakly fluorescent PSI. Therefore, we will briefly discuss these two processes separately.

### 4.3. Non Photochemical Quenching

NPQ is observed as a quenching of chlorophyll fluorescence determined by increased energy dissipation as heat and whose extent increases progressively with the intensity of the incident light. Therefore, it is considered principally as a strategy to down-regulate the efficiency of PSII light harvesting under conditions in which the incident photon flux exceeds the maximal utilisation capacity by the thylakoid electron transfer chain. *In vivo*, NPQ is a complex process that depends on several factors, which are often not completely independent one from the other. It has become customary to differentiate the processes that contribute to the overall NPQ process on the basis of their kinetics of formation and, even more frequently, of relaxation [10, 366-368].

*High-energy quenching (qE).* The prominent and fastest component of NPQ is called “high energy state quenching” (abbreviated as qE). This component, which is strictly dependent on the *energization* of the thylakoids by acidification of the luminal compartment [10, 359, 360, 366, 369, 370], is characterised by relatively rapid relaxation kinetics when the actinic illumination is turned off (in the order of ~1-2 min). Acidification of the lumen is responsible for at least two factors determining the onset of qE: 1) it activates the thylakoid-bound violaxanthin de-epoxidase enzyme (VDE), a crucial enzyme of the so-called xanthophyll cycle [287, 360, 371, 372], able to convert violaxanthin to zeaxanthin [372]; 2) it activates the PsbS protein by protonation of two lumen-exposed glutamate residues [373]. PsbS is a particular product of the Lhc gene superfamily with four transmembrane helices that is necessary for fast (tens of seconds to a few minutes) qE activation [374], even though slow qE formation in the absence of PsbS has been more recently reported (about half an hour) [375]. The detailed molecular mechanisms that give rise to qE are still a matter of intense debate. Most of the experimental evidences and proposed models suggests that qE occurs at the level of Lhcb proteins [10, 376, 377], and that some conformational changes in PSII antennas are essential for qE [142, 144, 279, 378-381]. It has been also suggested that these changes are regulated by PsbS, in a manner that still needs to be elucidate, and modulated by the binding of zeaxanthin to the Lhcb antennas [134, 359, 361, 382]. For instance, it has been shown that PsbS affects the rigidity of grana membranes and the readjustment of the antenna organization that might result in the formation of quenching sites [93-95]. The role of zeaxanthin has been much debated [10, 134, 359-361]. Although it is clear that the concentration of zeaxanthin increases at the expenses of violaxanthin when leaves are illuminated by high light [360], qE was shown to develop in thylakoid in the absence of the xanthophyll cycle [383-385], albeit with different kinetics of formation and relaxation kinetics. It was therefore proposed that zeaxanthin acts indirectly as an allosteric modulator of qE [10, 376], probably controlling the organization of the antenna complexes and stabilising a “dissipative” conformational state of the complexes. Both monomeric Lhcb complexes [128, 137, 279, 380, 386-388] and trimeric LHCII [142, 147, 389-395] have been proposed as the sites where the quencher is formed. In LHCII the quenching centre was proposed as originating from Chl-Chl interactions [10, 142, 144, 393, 395, 396]. This suggestion was supported by the evidence that low energy states emitting at ~700 nm can be induced in isolated Lhcb complexes upon aggregation *in vitro* [10, 142, 144, 393, 395, 396] and that similar fluorescence changes can be observed also *in vivo* at low temperature [144]. However, whereas aggregation quenching of Lhcb protein *in vitro* is well established, there is no clear evidence that the ~700 nm energy states, although present, have a low quantum yield [380, 381], which is a requisite for them being quenching centres. Still, they might represent a spectroscopic marker for conformational reorganisation of light harvesting complexes architecture in PSII [359, 396]. Zeaxanthin was also proposed to have a direct role in the quenching of chlorophyll excitation either through direct energy transfer from the chlorophyll excited singlet state to the zeaxanthin low lying S_1_ state, which decays rapidly, hence acting as a quencher [142, 397, 398], or through the energy transfer to a [Chl-Zea “couple”] possessing a strong charge-transfer character and that would act as quencher by the electron-transfer mechanism by forming a [Chl^–^/Zea^+^] charge separated state [399-401]. Recently, it has been proposed that the quenching by the Chl^–^/Zea^+^ charge separated state mechanism is likely to represent a particular component of NPQ, which was therefore called qZ for zeaxanthin-dependent quenching [134, 137]. Interestingly it was also shown that singlet excited state quenching can occur by the formation of Chl^–^/Lutein^+^ state, although probably occurring at different sites within the Lhca complexes [402], but still raising the question whether the mechanism is strictly dependent on zeaxanthin. For instances theoretical calculation of the energy levels indicate that violaxanthin might be also involved in a similar mechanism, as well as in the energy transfer mechanism but in both cases with lower efficiency, because of the shorter chain of conjugated double bonds [403, 404]. Another mechanism that has been proposed, particularly for the case of LHCII, is direct quenching of Chl singlet excited state by the low lying (S_1_) singlet state of lutein [144, 398]. As indicated above, also the site/s where the quenching occurs is under debate, some authors favour a major role of the minor complexes in qE, and others suggest the prominent role of the major LHCII trimeric complexes. Irrespective of the location of the quenching centres and the exact molecular mechanisms by which excited state quenching occurs, all models propose a change in interactions between the bound pigments, being either Chl-Chl or Chl-Car, which would promote the formation of a quenching centre. Such modification in the pigment-pigment interactions is thought to be associated with conformation of the protein structure, leading to alteration of either the inter-chromophore distances or mutual orientations, i.e., the factors that determine the interaction energy. The analysis by thermodynamic methods, monitoring quenching in isolated LHCII either as a function of pressure [378] or temperature [379] as well as single molecule analysis [380, 381] suggests that the complex can be in equilibrium between a “quenched” and an “unquenched” state and that the transition is kinetically limited by the activation barrier [379, 405]. Still, there is, to our knowledge, no evidence that a similar equilibrium between LHCII states occurs also *in vivo,* so that further evidence is needed to substantiate the hypothesis.

Another factor that has been suggested to be important for NPQ is the control of the ordered organisation in the PSII in the grana membranes, which gives rise to a chiral macrostructure (often referred to a “macrodomain”) that can be observed by circular dichroism spectroscopy. The observation of a light-induced decrease in the size of these chiral macrodomains, which correlates with the light-induced fluorescence quenching, has been reported and interpreted in terms of a reorganisation of the membrane structure possibly leading to the monomerisation of a population of LHCII trimers [406-409].

It is clear that even in the presence of an increased body of information, the details of the process still need elucidation. Whether it is not unlikely that all the proposed mechanisms participate somehow in the onset of NPQ, some might be or become predominant depending on the illumination and/or the physiological conditions of the organelle.

Whereas it is generally accepted that the regulative photoprotective role of PsbS is limited only to energy dissipation in PSII and it has no function on PSI, the role of zeaxanthin in photoprotection is more articulated. As indicated above, zeaxanthin protects PSII participating in qE and qZ activation by reversible binding to Lhcb proteins in a yet partially not understood mechanism. Zeaxanthin free in the membrane is also important for ROS scavenging [410, 411] and this function is useful in preventing damages both to PSII and PSI. Binding of zeaxanthin to PSI under high light conditions has also been reported [129, 133], but the precise function is not clear. However, recent results [133] points out a role of zeaxanthin in the modulation of ^3^Chl* yield when bound to both Lhcb and Lhca complexes, thus suggesting a functional role of zeaxanthin bound to PSI antennas.

*Sustained or inhibition-associated quenching (qI).* The most slowly reverting component of NPQ is called qI for photoinibitory quenching: *in vivo* qI recovers with half times of the order of several hours, thus comparable with those of PSII turnover, whereas *in vitro* it is substantially irreversible. In the case of qI, the nature of the quencher has been less intensively investigated and therefore remains to be elucidated. Yet, qI has been related to the capacity of non-functional (closed) reaction centres of PSII to dissipate the excess light energy (the so called RC quenching) [412]. However, the protective effect of qI with respect to photoinibition appears to be modest [413].

*State Transitions. *An important photosynthesis regulation that also manifests itself as a change in the intensity of Chl fluorescence emission, are the “state transitions” [363]. This phenomenon consists in the redistribution of excitation energy between PSI and PSII depending on the association of a mobile LHCII with PSII (State 1) or PSI (State 2). The association of LHCII in state 2 to PSI, which has a very low fluorescence yield, is detected as a decrease in total leaf fluorescence yield. Therefore, often, state transitions are treated as a component of the NPQ, called qT [147], characterised by an intermediate recovery time of ~5 min. Still, state transitions, rigorously speaking, are not a pure quenching process, but rather a process that modulates the absorption cross section of PSII (the emission of which is dominant with respect to that of PSI, at least at F_M_ induced by a saturating flash). Hence considering state transitions as a component of NPQ is a very rough approximation that is typically underestimated when analysing the experimental results. When PSII is preferably excited (State 2), LHCII is phosphorylated [88, 414, 415] and moves towards the unstacked region of thylakoids where PSI is located (Fig. **1A**). On the contrary, when PSI is preferentially excited (State 1), LHCII is dephosphorylated and migrates back to PSII [88]. State transitions are principally observed under non saturating light conditions [88], where the redistribution of the antenna cross-section between the two photosystem can have a significant effect in increasing the overall thylakoid ET rate [416]. In higher plants the size of the mobile LHCII has been quantified in about 20-25% of the total LHCII pool [88], whereas much pronounced reorganization, up to 80%, was observed in green algae where this process is associated with the switch between linear and cyclic electron flow (for detailed reviews on this topic, see [364, 365]).

In *Arabidopsis thaliana*, the STN7 kinase is responsible for the phosphorylation of LHCII [417]. In State II, the plastoquinone pool becomes more reduced (because of the light-limited turnover of PSI). When reduced PQ is bound to the *Q_0 _*site of the Cyt *b*_6_*f*, this leads to a conformational change in this complex [418] that activates STN7. It has been suggested that the phosphorylation at the N-terminus of LHCII causes a conformational change that lowers the affinity of LHCII for PSII and at the same time increase the affinity for PSI [419]. In State I instead, a thylakoid peripheral protein (TAP38/PPH1) [420, 421] dephosphorylates LHCII upon which it migrates back to PSII. Analyses of different PSI mutants showed that the PsaH subunit is essential for the docking of LHCII [422], but also other subunits are important (for instance PsaL, PsaO and probably PsaP) for the formation of the interaction. Recently, a protocol for stable isolation of PSI-LHCII has been developed [48] and allowed some characterization of this supercomplex. It has been found that the mobile trimers involved in state transitions are mainly the L trimers loosely bound to PSII, which are enriched in particular Lhcb1/2 isoforms. Interestingly, the strength of the binding of L trimers to PSI and the rate of excitation energy transfer to PSI are higher than the same processes when this LHCII is bound to PSII [48]. State transition are considered a regulatory process acting at relatively low light, since at high light energy dissipation as qE is considered more important than excitation balancing between photosystems. However, recent investigations [150] confirmed previous literature reports [149] indicating that LHCII can bind to PSI even under high light. All these findings suggest that a part of the LHCII trimers might be considered an intimate part of the PSI antenna system which moves to PSII under State I conditions [48].

### 4.4. Efficiency of Singlet Excited State Quenching in Preventing Photoinhibition

Whereas the role of state transition is mainly discussed in terms of optimisation of light harvesting under limiting light conditions, the role of NPQ is generally associated with a protective strategy against photo-inhibition. The general idea is that by increasing thermal dissipation and lowering the Chl singlet excited state population there is a proportional decrease in the ^3^Chl^*^ populated by intersystem crossing and also a lower yield of ^3^P_680_* (or other potentially harmful species formed during the catalytic turnover of PSII), because of the reduced probability of energy transfer from the antenna to the RC. Yet, in a series of studies in which the effect of excited state quenching induced by artificial quinone quenchers was investigated systematically, both in intact cells [314] and in isolated thylakoids [269, 313], it was shown that the rate of net photo-inhibitory damage is not affected to a great extent by this quenching, with protection estimated to be ≤ 30%, which is much less than that expected on the basis of the linear-dose response relation between light intensity and photoinhibitory damage [269, 313-315, 337, 423]. Moreover, photoinhibition in thylakoids isolated from some NPQ mutants appears to be at the same level as the wild-type [424]. On the other hand, the *in vivo* analysis of mutants with reduced NPQ shows that photoinhibition is enhanced [425-427]. The overall increased sensitivity of NPQ deficient mutants, as in the *npq4* mutant lacking PsbS, is apparent especially when plants are grown in the natural environment, whereas the effect is smaller when plants are grown under controlled conditions [428]. The NPQ deficient/impaired mutants were also shown to have a lower “fitness” producing ~30% less seeds than the control plants under natural conditions [428]. Thus, there appears to be contradictory results obtained either *in vivo* or *in vitro*. However, in the *in vitro* studies (or *in vivo* with inhibited protein turn-over), the analysis was specific for the direct damage induced by high light stress, whereas *in vivo* it involved necessarily the whole plant metabolism. Such discrepancies might then be hypothetically reconciled, considering that PSII turnover is a particularly costly metabolic process, and that for long time scale experiments, as those typically performed *in vivo* that require several hours of illumination, and in certain cases even days of exposure to fluctuating ambient light conditions (whereas inhibition is commonly observed in less than one hour *in vitro*, depending on the light intensity used in the experiments), even what might appear as a moderate protective effect (20-30%) could be relevant on the whole metabolic scenario. Moreover, NPQ has also the effect of modulating (lowering) PSII quantum efficiency as a function of light intensity, a process that is also likely to contribute to the balance of electron flow through the thylakoid membrane, which is the principal source of ATP production in the leaves, avoiding, for instance, that the PQ remains in an almost fully reduced state under saturating light regimes.

## CONCLUDING REMARKS

In this review we have illustrated some features of the plant photosystems, which are highly organised pigment protein supercomplexes that convert light energy into chemical energy and sustain life on earth. The two photosystems bind similar cofactors, have some similar subunits and are exposed to the same environment and therefore they share many common macroscopic properties. However, due to the necessity to catalyse different reactions, during evolution the properties of cofactors bound to the two photosystems have been tuned in order to be able to work in series. PSII and PSI are the enzymes that probably operate at the most oxidising (PSII) and the most reducing (PSI) redox potentials in nature.

Despite the fact that much very detailed information is now available concerning the mechanism of excitation energy transfer and electron transfer in plant photosystems, many details about how these supercomplexes attain their outstandingly maximal photochemical efficiencies, which always exceed 80%, are still unknown. Moreover, the detailed mechanisms leading to the regulation of the photosystem activity under natural fluctuating light environments, particularly at the level of the entire supercomplex, remain to be elucidated.

A high-resolution structure of plant PSII is not available yet, due to the difficulty in the purification of intact PSII caused by the weak connections between subunits and the well-known complexity associated with the crystallisation of large membrane proteins. There are indications that the cyanobacterial PSII core may not be a sufficiently good model for in depth investigations on plant PSII. Therefore a structural model of plant PSII would be of inestimable value for the understanding of PSII functioning as well as for the interpretation of biochemical and spectroscopic data. In the case of PSI, the crystallographic structure has been of central importance for the interpretation of biochemical and spectroscopic result obtained both previously and successively to the resolution of its crystallographic model. Yet, different PSI domains are still at low resolution, as the region of the Lhca proteins (containing the red forms of PSI) or the region which also acts as the docking site for LHCII, which probably contains the PsaO and PsaP subunits, thus preventing the full description of PSI function. Moreover, the function of several other small subunits both of PSI and PSII remains unknown or is only partially understood.

Photosynthesis is probably one of the most multidisciplinary fields of research. The synergy of many approaches, such as genetics, genomics, molecular biology, biochemistry, biophysics, spectroscopy, proteomics and physiology has been of crucial importance in elucidating the properties of both photosystems as well as their physiological role at the organelle and whole organism level. The continuous development of such multidisciplinary approaches will be necessary to improve the understanding of the functionality and to shed light on the yet unknown characteristic of these extremely efficient natural energy converters.

## Figures and Tables

**Table 1.** Pigment content of Lhc antenna complexes and photosystems.

	Chl a	Chl b	tot Chls	Chls a/b ratio	tot Cars	Neo	Vio	Lut	β-car	Ref	Notes
PSII core	35 (RC:6; CP47:16; CP43:13)		35		11-12				11-12	[19, 20]	Data from cyanobacterial cores (see text for possible differences with plant PSII).
LHCII (monomeric Lhcb1-2-3)	8	6	14	1.33	4	1	0.5	2.5	-	[122, 129]	Different isoforms may have slightly different pigment contents (see text).
CP29 (Lhcb4)	8.5	4.5	13	1.90	3	1	0.8	1.2	-	[124, 131]	
CP26 (Lhcb5)	9	4	13	2.25	3	1	0.5	1.5	-	[135]	Chl amount estimated by sequence homology.
CP24 (Lhcb6)	6	5	11	1.20	2	-	1	1	-	[128, 429]	
PSII (C2S2M2)	~220	~100	~320	~2.2	~86	~16	~11	~37	~22	calculated	Largest purifiable PSII supercomplex.
PSII (C2S2M2L4)	~320	~170	~490	~1.9	~134	~28	~18	~66	~22	calculated	Largest PSII in membrane.
Lhca1	8.8	2.2	11	4.0	2.9	-	1.2	1.7	-	[53]	Refolded complexes (likely missing some Chls)
Lhca2	7.1	3.9	11	1.8	2.2	-	0.5	1.7	-	[53]	Refolded complexes (likely missing some Chls)
Lhca3	9.4	1.6	11	5.9	3.3		0.8	1.9	0.6	[53]	Refolded complexes (likely missing some Chls)
Lhca4	7.8	3.2	11	2.4	2.3	-	0.4	1.9	-	[53]	Refolded complexes (likely missing some Chls)
Lhca1-4 dimer	22	6	28	3.7	6.00	-	1.5	3.3	1.2	recalculated from [115]	Native dimer
Lhca2-3 dimer	22	6	28	3.7	6.00	-	1.1	3.0	1.9	recalculated from [115]	Native dimer
PSI	~155	~18	~173		~34	-	~3	~9	~22	[17, 48]	Few Chls could miss in the crystal structure (and so some Cars since calculated on the Chls/Cars ratio)

Stoichiometries have been obtained by crystal structures and biochemical data. Note that small differences could exist between the same complexes from different species. Neo: neoxhanthin; Vio: violaxanthin, Lut: lutein; β-car: β-carotene.

## ABBREVIATIONS

Car: = Carotenoid
Chl: = Chlorophyll
ET: = Electron Transfer
ISC: = Intersystem Crossing
Lhc: = Light harvesting complex
Lhca (or LHCI): = Light harvesting complexes of PSI
Lhcb: = Light harvesting complexes of PSII
Lhcb4 (CP29), Lhcb5 (CP26), Lhcb6 (CP24): = Monomeric Lhc of PSII
LHCII: = Major Light Harvesting Complex of PSII
NPQ: = Non Photochemical (energy) Quenching
Pheo: = Pheophytin
PhQ: = Phylloquinone
PQ: = Plastoquinone
PSI, PSII: = Photosystem I and II, respectively
Q_A_,Q_B_: = Plastoquinone at the site Q_A_ and Q_B_ in PSII, respectively
RC: = Reaction Centre
ST: = State Transitions
